# Review of pyronaridine anti-malarial properties and product characteristics

**DOI:** 10.1186/1475-2875-11-270

**Published:** 2012-08-09

**Authors:** Simon L Croft, Stephan Duparc, Sarah J Arbe-Barnes, J Carl Craft, Chang-Sik Shin, Lawrence Fleckenstein, Isabelle Borghini-Fuhrer, Han-Jong Rim

**Affiliations:** 1 Faculty of Infectious and Tropical Diseases, London School of Hygiene and Tropical Medicine, Keppel Street, London, WC1E 7HT, UK; 2 Medicines for Malaria Venture, Geneva, Switzerland; 3 Aptiv Solutions, Stevenage BioScience Catalyst, Stevenage, SG1 2FX, UK; 4 Former MMV Chief Scientific Officer, Libertyville, IL, USA; 5 Shin Poong Pharmaceuticals Co Ltd, Seoul, Republic of Korea; 6 College of Pharmacy, University of Iowa, Iowa City, IA, USA; 7 Department of Parasitology, College of Medicine, Korea University, Seoul, Korea

**Keywords:** Pyronaridine, *Plasmodium falciparum*, *Plasmodium vivax*, Review, Artemisinin containing compound, Anti-malarial therapy

## Abstract

Pyronaridine was synthesized in 1970 at the Institute of Chinese Parasitic Disease and has been used in China for over 30 years for the treatment of malaria. Pyronaridine has high potency against *Plasmodium falciparum*, including chloroquine-resistant strains. Studies in various animal models have shown pyronaridine to be effective against strains resistant to other anti-malarials, including chloroquine. Resistance to pyronaridine appears to emerge slowly and is further retarded when pyronaridine is used in combination with other anti-malarials, in particular, artesunate. Pyronaridine toxicity is generally less than that of chloroquine, though evidence of embryotoxicity in rodents suggests use with caution in pregnancy. Clinical pharmacokinetic data for pyronaridine indicates an elimination T_1/2_ of 13.2 and 9.6 days, respectively, in adults and children with acute uncomplicated falciparum and vivax malaria in artemisinin-combination therapy. Clinical data for mono or combined pyronaridine therapy show excellent anti-malarial effects against *P. falciparum* and studies of combination therapy also show promise against *Plasmodium vivax*. Pyronaridine has been developed as a fixed dose combination therapy, in a 3:1 ratio, with artesunate for the treatment of acute uncomplicated *P. falciparum* malaria and blood stage *P. vivax* malaria with the name of Pyramax® and has received Positive Opinion by European Medicines Agency under the Article 58 procedure.

## Background

Pyronaridine (4-[(7-chloro-2-methoxybenzo*b*[1,5]naphthyridin-10-yl)amino]-2,6-bis[(pyrrolidin-1-yl)methyl]phenol) [Recommended INN: List 59 41: WHO Drug Information, Vol. 22, No. 1, 2008] is a benzonaphthyridine derivative first synthesized in 1970 at the Institute of Chinese Parasitic Disease, Chinese Academy of Preventative Medicine [1,2]. Pyronaridine also referred to as ‘7351’ and Malaridine® has been used in China for the treatment of malaria as a single agent for the past 30 years.

More recently, interest has been renewed in pyronaridine as a possible partner for use in artemisinin-based combination therapy (ACT) for malaria treatment. Indeed, its limited use outside China suggests that resistance will be slow to emerge when used in ACT and pyronaridine is currently under investigation as a fixed-dose combination administered in a 3:1 ratio with artesunate for the treatment of uncomplicated *Plasmodium falciparum* and blood stage *Plasmodium vivax* malaria. This review examines the *in vitro* activity, anti-malarial effect, toxicology, pharmacokinetic and clinical data on pyronaridine.

## Chemistry, presentation and properties

**Figure 1:**
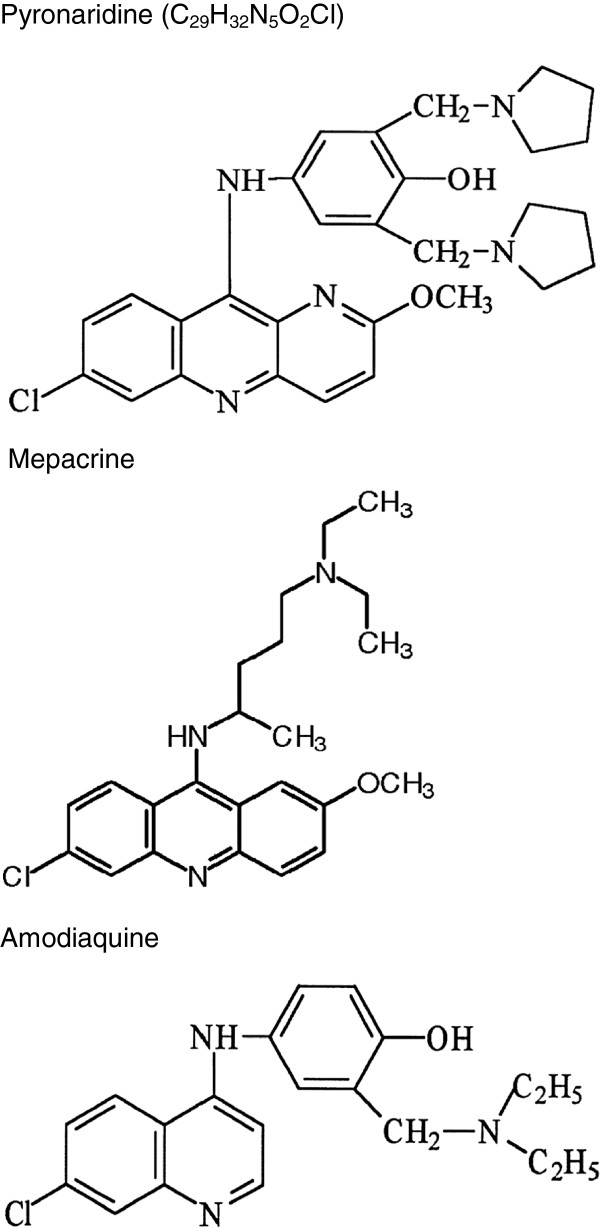
**Comparative activity of pyronaridine against chloroquine-susceptible and -resistant* P. falciparum *field isolates from SE Asia and Africa.**

The pyronaridine nucleus is based on mepacrine (a 9-aminoacridine) with the addition of an amodiaquine-like side chain (Figure 1) [3,4]. The drug is administered as pyronaridine tetraphosphate (56.89% base), a yellow, odourless powder with a bitter taste [3]. The drug was produced in China as tablets for oral use, as an injectable liquid for intramuscular (IM) administration and has also been administered intravenously (IV) [3,5]. Almost all published clinical trials to date used the Chinese enteric-coated tablet formulation with 175 mg of the tetraphosphate, equal to 100 mg base, with dosages calculated as the free base. The Chinese also developed a capsule formulation available as 100 mg and 50 mg free base. A capsule fixed-dose formulation of pyronaridine tetraphosphate plus artesunate (3:1 ratio) has been reported by University Sains, Malaysia with pyronaridine dosages based on the tetraphosphate [6]. 

Pyronaridine free base is ‘very sparingly’ soluble in water, whereas the tetraphosphate salt is ‘sparingly’ soluble in water (1.46%), aiding oral absorption [7]. The greater hydrophobicity index (Rm 0.872) of the salt confirms the higher aqueous solubility *versus* the base (Rm 0.773). Pyronaridine has been found to be highly lipophilic at pH 7.4 (logD 0.34); lipophilicity was reduced at pH 5 [8]. The base is more liposoluble than the salt [7].

## *In vitro* studies

Pyronaridine has potent *in vitro* activity against *P. falciparum* strains [9-13] and clinical isolates [9,10,12,14-17] including those that are resistant to other anitmalarials. The mechanism(s) by which pyronaridine acts as an antimalarial has been examined. Initial studies demonstrated that pyronairidne interfered with the digestive system of *P. falciparum* and *Plasmodium berghei*[18]. Subsequently it was reported that pyronaridine inhibited production of, and formed complexes with, β-haematin to enhance haematin-induced human blood cell lysis [19,20]. Pyronaridine also inhibits glutathione-dependent haem degredation [19,21].

*In vitro* combination of pyronaridine with other antimalarial agents including artesunate or DHA has shown either additive effects [22] or weak antagonism [11,23,24], dependent on the experimental model employed.

## Mechanism of action

Early studies indicated that pyronaridine appeared to interfere with the food vacuole of the parasite [25,26]. In erythrocytic *P. falciparum* and *P. berghei* cultured *in vitro* in human erythrocytes, pyronaridine induced modifications to the food vacuoles followed by the rapid formation of multilameliate whorls in the pellicular complexes of trophozoites [18]. Similarly, ultrastructural analysis of *P. falciparum* after pyronaridine treatment of infected primates (*Aotus trivirgatus*) showed the earliest and most distinct effect of therapy was on the parasite food vacuole of late trophozoites and schizonts; specifically, undigested endocytic vesicles surrounded by a single membrane in the vascular space were observed [27].

Subsequent *in vitro* studies have reported that pyronaridine targets haematin formation [19,20,28]. Pyronaridine inhibited β-haematin production with an IC_50_ similar to that of chloroquine (0.125 μM) and formed complexes with β-haematin with a 1:2 stoichiometry to enhance haematin-induced human blood cell lysis. 10 μM of pyronaridine was needed for complete lysis, approximately 1/100 of the concentration needed with chloroquine [29]. However, there was no clear evidence for antagonism between pyronaridine and other anti-malarials that target haematin formation (chloroquine, mefloquine or quinine) [29]. Pyronaridine has also been shown to inhibit glutathione-dependent haem degredation [19,21].

Another study reported that pyronaridine inhibited the decatenation activity of *P. falciparum* DNA topoisomerase II [30]. However, an assay detecting the presence of protein−DNA complexes within parasite cells found no inhibitory effect of pyronaridine against * P. falciparum * topoisomerase II activity *in situ*[31].

## *In vitro* activity against *Plasmodium falciparum*

Using various techniques, there is a great deal of evidence that pyronaridine has potent *in vitro* activity against *P. falciparum* clinical isolates (Additional file 1) [9,10,14-17], and strains (Additional file 2) [9-13,32], including those resistant to other anti-malarials.

Pyronaridine activity against erythrocytic *P. falciparum* is greatest for the ring-form stage (ED_50_ 8.3 [95% CI 8.1−8.4] nM), followed by schizonts (11.6 [11.4−11.9] nM) then trophozoites (14.0 [13.4−14.7] nM) [33]. Pyronaridine was more active against all of these stages than chloroquine: ED_50_s for ring-forms, schizonts and trophozoites were 24.5 (24.3−24.8), 64.9 (58.1−72.4) and 34.0 (32.4−35.6) nM, respectively [33].

Pyronaridine retains high activity against chloroquine resistant strains. For example, an *ex vivo* study of serum from *Saimiri* monkeys given 30 mg/kg pyronaridine gave an IC_50_ of 7 ± 5 ng/ml and an IC_90_ of 11 ± 9 ng/ml against the multi-drug resistant K1 *P. falciparum* strain [34]. In comparison the IC_50_ for chloroquine was 107 ± 36 ng/ml and the IC_90_ 152 ± 46 ng/ml, and values for amodiaquine were 9 ± 3 ng/ml and 10 ± 4 ng/ml, respectively. Pyronaridine activity was notably more prolonged than either chloroquine or amodiaquine in this study [34].

Moreover, the maximal effect of pyronaridine appears to be unaffected by chloroquine resistance, pyronaridine E_max_ (% inhibition) was 95−100% against three chloroquine-sensitive strains, comparable to the maximal inhibitory activity of artemisinin (97−100%), dihydroartemisinin (96−99%) and artemether (87−97%). Against a chloroquine-resistant strain (D7, Tanzania) pyronaridine E_max_ was 96 ± 7% compared with 84−91% for the artemisinins [13].

## Cross resistance with chloroquine and other anti-malarials

The activity of pyronaridine against chloroquine sensitive and resistant field isolates and strains is shown in Additional files 1 and 2[9-17,32]. Structurally related compounds to pyronaridine, such as amodiaquine, demonstrated similar activity against chloroquine sensitive and resistant strains [17]. Note that the cut-off value for *in vitro* reduced susceptibility to pyronaridine has not been validated and all cut-offs reported in the literature are arbitrary [14,16].

Early studies of isolates from Hainan (FCC-4/HN, FCC-5/HN), Anhui (FCC101/AH), and Jiangsu (FCC-102/JS) provinces in China and from Cambodia (Camb-I) showed no evidence of cross resistance between pyronaridine and chloroquine: the pyronaridine IC_50_ was 12.4−22.6 nM against chloroquine-sensitive strains *versus* 10.9−17.6 nM against chloroquine-resistant strains [35]. Pyronaridine was also significantly more active against the K1 (Thailand) chloroquine-resistant strain than against a chloroquine-sensitive one (F32 [Tanzania]) (*P* < 0.05) [36].

In one study of field isolates from eastern and northern Thailand, no cross resistance was found between pyronaridine and chloroquine, quinine, amodiaquine, pyrimethamine or mefloquine (Additional file 1), though there was some degree of cross resistance between pyronaridine and chloroquine for reference strains (Additional file 2) [9]. Another study conducted in Thailand found a statistically significant correlation between chloroquine and pyronaridine IC_50_ and IC_90_ (*r* = 0.447, *P* = 0.0006 and *r* = 0.477, *P* = 0.0002, respectively) [37]. However, a statistically significant result (*P* < 0.05) can often been seen when comparing drug activities against large numbers of isolates; when looking at correlations, an *r* value of > 0.7−0.8 or *r*^*2*^ of > 0.5 is more suggestive of cross resistance. Considering this, the correlations between pyronaridine and chloroquine IC_50_ and IC_90_ in this study were weak. In a study of the uptake of [3 H] pyronaridine by *P. falciparum*-infected erythrocytes, uptake was similar for both chloroquine-sensitive and chloroquine-resistant strains (T9.96 and K1), being five times less for the comparator chloroquine [38].

An analysis of African field isolates found no correlation between the *in vitro* activity of pyronaridine and chloroquine or monodesethylamodiaquine, though there was a correlation with amopyroquine (Additional file 1) [10]. Surveillance studies of 67 *P. falciparum* infections imported from East and West Africa into Italy showed 33.3% pyronaridine resistance and a weak correlation with chloroquine resistance (*r* = 0.38, *P* < 0.05) [39]. Surprisingly, chloroquine resistance was only 14% in these strains [39]. The relevance of these findings is unknown as they do not appear to reflect the situation reported for transmission zones.

Data are also available from individual countries in Africa. A study in Cameroon found no evidence of cross resistance between pyronaridine and chloroquine (Additional file 1) [17]. Similarly, a recent study in Cameroon found high levels of susceptibility to pyronaridine (89.5%) in all isolates, including chloroquine-resistant isolates [40]. In contrast, field isolates from Senegal showed a weak correlation between pyronaridine and chloroquine *in vitro* activity (*r* = 0.44, *P* < 0.001) (Additional file 1) [16]. Pyronaridine activity was significantly decreased for chloroquine-resistant (4.9 nM) *versus* sensitive strains (2.9 nM, *P* < 0.002), though pyronaridine was around 12-fold and 50-fold more active than chloroquine against these strains, respectively [15,16]. Similarly, in field isolates from Gabon there was a strong correlation between pyronaridine *in vitro* activity and that of amodiaquine and halofantrine (*r* = 0.74, *r* = 0.71, respectively, *P* < 0.001 for both) with a weaker correlation for quinine, and chloroquine (*r* = 0.60, *r* = 0.51, respectively, *P* < 0.001 for both) [14]. The IC_50_ for pyronaridine was ≤ 4.3 nM in all strains tested (n = 59) (Additional file 1) [14]. Pyronaridine and chloroquine IC_50_ against 38 clinical isolates from Mogadishu, Somalia also showed a significant but weak correlation (*r* = 0.5733, *P* = 0.001) [41].

A study of 12 reference strains/clones from various regions found a correlation between pyronaridine and chloroquine at the IC_50_ level (*r* = 0.63, *P* = 0.03), but not at the IC_99_ level (*r* = 0.54, *P* = 0.07) [32]. Contrastingly, the opposite was found for pyronaridine and quinine (IC_50_*r* = 0.48, *P* = 0.12; IC_90_*r* = 0.71, *P* = 0.01) [32]. There was no cross resistance with mefloquine, but there was with mepacrine and amodiaquine at the IC_50_ (*r* = 0.87, *P* = 0.01; *r* = 0.95, *P* = 0.01, respectively) and IC_99_ (*r* = 0.85, *P* = 0.01; *r* = 0.77, *P* = 0.01, respectively) (Additional file 2) [32].

Thus, the relationship between pyronaridine and chloroquine susceptibility in *P. falciparum* appears to be more complex than simple cross resistance and may vary regionally. However, it is certain that pyronaridine is far more active than chloroquine against *P. falciparum in vitro* and retains high activity against the majority of chloroquine-resistant field isolates (Additional file 1) [9,10,14-17], and reference strains (Additional file 2) [9-13,32].

## Combination therapy

Conflicting data exists concerning the *in vitro* effect of pyronaridine in combination with other anti-malarials. In one study, there was synergy between pyronaridine and primaquine, an additive effect with 4-aminoquinolines and weak antagonism with antifolates, amino alcohols and dihydroartemisinin [24]. Further studies also found weak antagonism between pyronaridine and dihydroartemisinin or artesunate [11,23]. However, both additive and synergistic effects between artemisinin and pyronaridine has also been reported [22]. These differences are probably methodological and *in vivo* studies indicate high efficacy and a synergistic effect between pyronaridine and artemisinin or artesunate against pathogens with *in vitro* resistance to these agents [11,42].

## Mechanism and development of resistance

The mechanism by which resistance to pyronaridine develops is unknown, but may be may be due to a direct effect on the pyronaridine mechanism of action. Cross resistance with chloroquine *in vitro* appears to be incomplete and inconsistent, whereas, *in vivo* pyronaridine retains activity against chloroquine-resistant strains. Thus, there may be important differences between chloroquine and pyronaridine resistance and more than one resistance mechanism may operate in order to overcome the high potency of pyronaridine against *P. falciparum*.

The activity of pyronaridine against chloroquine-sensitive and -resistant field isolates from China and Cambodia showed no evidence of cross resistance between pyronaridine and chloroquine [11]. Pyronaridine was also reported to be significantly more active against a chloroquine-resistant strain from Thailand compared with a chloroquine-sensitive strain from Tanzania [32].

Wu *et al.*[43] described an increase in the number of food vacuoles in trophozoites from a pyronaridine-resistant *P. berghei* (RP) line, some of which were fusing. There was also a marked reduction in the digestive food vesicles containing malaria pigment granules for both trophozoites and schizonts and typical haemozoin grains were not formed in the pyronaridine-resistant parasites [43]. These and other ultrastructural differences suggested that resistance may be due to a direct effect on the pyronaridine mechanism of action.

Li *et al.*, found over-expression of a 54 kDa protein in a pyronaridine-resistant strain of *P. berghei* (ANKA) [44]. The protein was localized mainly in the cytoplasm of erythrocytic stage trophozoites, schizonts and merozoites and less commonly in the cytoplasm of infected erythrocytes [44]. Interestingly, a 54 kDa protein is also expressed in chloroquine-resistant *P. berghei* (ANKA) suggesting a common effect, though whether this is related to resistance development *per se* is unknown [44].

Shi *et al.* described the relationship between pyronaridine resistance and the alteration of parasite polyamine metabolism [45]. Infection with a *P. berghei* (PRB) strain of high pyronaridine resistance significantly increased the concentrations of putrescine and spermidine *versus* a low pyronaridine resistant strain (PRA) by 1.8-fold and 1.7-fold and *versus* a pyronaridine-susceptible strain by 3.9-fold and 2.6-fold, respectively. Treatment of the pyronaridine-susceptible strain with 5 mg/kg pyronaridine decreased spermidine by 31.6% and spermine by 47.3%. In contrast, there was no change in polyamine levels in erythrocytes from mice infected with the highly pyronaridine-resistant strain when treated with 10 mg/kg pyronaridine [45].

## *In vitro* resistance

One early study found that resistance in *P. berghei* (ANKA) developed slowly to pyronaridine administered at 4 mg/kg, with no detectable high resistance within 31−45 passages [46]. However, higher doses were more effective at selecting for resistance [46]. More recently a study using 4.7 mg/kg pyronaridine recorded the development of resistance in *P. berghei* by the 15^th^ passage with an increase in ED_90_ from 0.8 to 4.4 mg/kg, though by the 35^th^ passage a peak ED_50_ of around 13.8 mg/kg was achieved and this did not increase with up to 50 passages [47].

The stability of pyronaridine resistance was also studied. Pyronaridine resistance established in *P. berghei* after 50 passages was mostly maintained after transfer through mice for five passages [47]. However, another study reported greater instability of pyronaridine resistance [48]. Here, pyronaridine-resistant *P. berghei* achieved after 35 passages (resistance >299 fold) was passed through mice given intragastric pyronaridine 600 mg/kg over three days. After 49 passages, relief of drug pressure restored pyronaridine sensitivity after five passages. After 115 passages, pyronaridine sensitivity started to return after 21 drug-free passages. Pyronaridine resistance was stable only after 134 passages, with no decrease in resistance after 31 drug-free passages and with cross resistance to chloroquine [48].

## *In vivo* resistance with combination therapy

Concomitant dosing of pyronaridine 4 mg/kg, sulphadoxine 5 mg/kg and pyrimethamine 1 mg/kg led to the development of low level resistance to all three agents by passage 31−34 [46]. However, in the same number of passages, concurrent administration of pyronaridine 2 mg/kg with sulphadoxine 2 mg/kg or pyrimethamine 0.05 mg/kg prevented selection of pyronaridine resistance and resistance to the triple combination [46]. This was early evidence of the possibility for reducing resistance selection with pyronaridine in combination therapy.

Peters *et al.* were able to derive pyronaridine-resistant *P. berghei* and *Plasmodium yoelii* strains by *in vivo* serial passage, applying drug at 3 or 10 mg/kg [49]. However, resistance development was slow, taking 140 days to reach maximal levels and was more difficult to achieve with the higher dose [49]. Further experiments using the 2% relapse technique with the combination of pyronaridine (1 mg/kg at each passage) and artesunate (300 mg/kg at each passage) resulted in a marked delay in the development of resistance in *P. berghei versus* pyronaridine alone [50,51]. These results are encouraging as they indicate that pyronaridine plus artesunate may retard the development of resistance.

## Resistance in clinical isolates

Data from China, where pyronaridine has been used both as a monotherapy and in combination therapy for around 40 years, suggests that resistance develops relatively slowly, even in the presence of extensive chloroquine resistance.

An analysis of clinical cases from 1984−1985 (n = 36) and 1995 (n = 39) in Yunnan Province, China showed a decline in the *in vitro* susceptibility to pyronaridine (ID_50_ 13.0 nM in 1988 *versus* 40.0 nM in 1995) and an increase in the rate of 28-day recrudescence from 15.2% (5/33) to 37.5% (9/24) [52]. There was also an increase in the time to clear fever in patients treated with pyronaridine over this period from 32.7 ± 16.0 h to 56.2 ± 27.4 h, though the time to parasite clearance was similar (64.2 ± 22.9 h *versus* 55.3 ± 11.8 h, respectively) [52]. Although this was a small study, other reports show that *in vitro* resistance to pyronaridine in Yuxi Prefecture, Yunnan Province, China, had emerged (38.1%), but at a lower prevalence than for chloroquine (85.7%) and piperaquine (66.7%) resistance [53]. Similarly, isolates from the China−Laos border, south of Yunnan Province, had a resistance prevalence of 34.5% to pyronaridine, 97.0% to chloroquine, and 96.4% to piperaquine [54]. A more recent report showed that clinical isolates obtained from Hainan and Yunnan Provinces during the malaria transmission season showed a gradual decrease in pyronaridine sensitivity over time, with the mean drug concentration for complete *in vitro* inhibition of schizont formation raised by two to four-fold. However, the clinical therapeutic efficacy of pyronaridine remained satisfactory [55].

## Resistance in clinical isolates after combination therapy

Combination therapy appears to decrease the potential for pyronaridine resistance development. When pyronaridine−sulphadoxine−pyrimethamine (500/1,000/50 mg) was administered as a single oral dose as the exclusive treatment in the Diaoluo area in Hainan Province, susceptibility was maintained with a 100% cure rate over a five-year period from 1986 [56].

Though data are limited, there was no evidence of the development of resistance after unsuccessful treatment with pyronaridine tetraphosphate−artesunate combination therapy: the initial pyronaridine mean IC_50_ was 23.39 ± 12.71 nM *versus* 22.96 ± 11.54 nM after recrudescence in *P. falciparum* isolated from nine patients for whom paired data were available [57].

## Anti-malarial effect

Pyronaridine has been reported to have potent activity against the erythrocytic stages of malaria infection in mouse models [57,58]. Efficacy against *P. berghei* ANKA for intragastric pyronaridine [45] and against *P. yoelii* for subcutaneous pyronaridine [59] have been demonstrated in mice. Indeed, pyronaridine exhibits extended schizontocidal activity in mice [8], which was more prolonged when compared with chloroquine [60].

The schizontocidal activity of pyronaridine has been investigated in primate models of malaria infection. Efficacy against *P. inui* has been demonstrated in Rhesus monkeys [57] and in *P. cynomolgi* intragastric pyronaridine was reported to be less effective than chloroquine [61]. However, intravenous pyronaridine has been shown to be more effective than the same dose of chloroquine [62]. Efficacy has also been demonstrated for intramuscular pyronaridine in primates [57,62].

*In vivo* studies using pyronaridine with either artemisinin, artesunate or DHA showed synergy and restored efficacy against strains resistant to the individual components or impeded selection of resistance to these compounds [26,41,50] indicating its potential in delaying the selection and spread of resistance.

Initial *in vitro* data indicated strong gametocytocidal activity of pyronaridine against two multi-drug resistant *P*. *falciparum* isolates (KT1 and KT3) [63]. However, these findings were not supported by *in vivo* studies where no changes in gametocyte density or morphology up to 24 h after administration were observed [59].

## Murine models

### Effect in erythrocytic stages

Pyronaridine has proved highly effective against the erythrocytic stages of malaria infection in mouse models. Early work in mice infected with *P. berghei* (Day 0) and given pyronaridine on Day 3, found that the ED_50_ at Day 6 was 6.8 ± 1.4 mg/kg (n = 4) for oral administration and 4.97 ± 0.65 mg/kg (n = 7) for IM administration; corresponding values for chloroquine were 45.6 ± 6.3 mg/kg and 30.89 ± 5.8 mg/kg, respectively [3,64]. Thus, pyronaridine, after oral or IM administration was approximately 6.7-fold and 6.2-fold more active than chloroquine, respectively [3,64]. An ED_50_ of 2.7 (95%CI 2.2−3.3) was reported for intragastric pyronaridine *versus* 16.7 (12.8−21.7) mg/kg for chloroquine, 9.1 (10−14) mg/kg for sulphadoxine and 4.3 (2.6−7.1) mg/kg for pyrimethamine in mice infected with *P. berghei* ANKA, which is susceptible to these four drugs [46]. In fact, combination therapy with pyronaridine (10 mg/kg) and sulphadoxine−pyrimethamine (3 mg/kg) was no more effective in clearing parasitaemia than pyronaridine alone (*P* > 0.05) in mice infected with *P. berghei* (ANKA) [65]. Efficacy against *P. yoelii* for subcutaneous pyronaridine 30 mg/kg has also been demonstrated in mice where 5/5 treated mice had no parasites at Days 5, 6 and 12 *versus* 1/5, 0/5 and 0/5 for controls, respectively [66].

In addition, pyronaridine exhibits schizontocidal activity for a longer duration compared with chloroquine: at 1.47 x the ED_50_ (10 mg/kg) pyronaridine was suppressive against *P. berghei* infection when given six days before inoculation, whereas chloroquine at the same relative ED_50_ (67 mg/kg) needed to be given two days before inoculation [5,67]. Follow-up experiments showed that pyronaridine 10 mg/kg (3x ED_50_) given to mice up to three days before inoculation with *P. berghei* (ANKA) protected 10−50% of animals from infection, whereas a 3 mg/kg dose of sulphadoxine/pyrimethamine (2:1, 4x ED_50_) had no protective effect [59]. Further evidence for an extended schizontocidal effect of pyronaridine comes from a study in mice infected with *P. berghei* N [8]. Pyronaridine at concentrations of 25 μmol.kg^-1^ and above was completely effective in clearing parasites from blood by Day 2 (n = 6). There was some recrudescence of parasitaemia at Day 4 (< 20% of baseline), though this had decreased to zero by Day 28 with 5/6 mice surviving. In contrast, recrudescence in animals treated with amodiaquine occurred from Day 4 and all mice were dead by Day 14 [8].

### Effect against tissue schizonts

Studies in China found that pyronaridine (2.5 mg/kg) had no effect on the schizontocidal effect of primaquine against *P. yoelii* tissue schizonts in mice (n = 10) [59]. A later study confirmed that pyronaridine (10−100 nM) did not inhibit the growth of * P. yoelii * sporozoites in C57BL/6 mouse hepatocytes * in vitro *[66].

### Effect against drug-resistant strains

In mice, oral pyronaridine was found to be equally effective against chloroquine-sensitive and chloroquine-resistant *P. berghei*. The ED_50_ (n = 4/group) for three doses was 2.01 ± 0.19, 2.04 ± 0.26 and 2.61 ± 0.44 mg/kg for the sensitive *versus* 1.99 ± 0.27, 2.66 ± 0.36 and 2.88 ± 0.48 mg/kg for the resistant strain, giving a resistance index (resistant/sensitive ED_50_) of 0.99−1.11 [64].

However, attenuation of oral pyronaridine efficacy against a chloroquine-resistant strain has been reported [68]. Pyronaridine (intragastric) 25 or 50 mg/kg over four days cured 8/10 (recrudescence Day 24 and 31) and 10/10 mice infected with drug-sensitive *P. berghei* (ANKA), respectively; no mice died. Pyronaridine was more effective than amodiaquine (50 or 100 mg/kg/4 days), mefloquine (50 or 100 mg/kg/4 days) and artemisinin (200 or 400 mg/kg/4 days) [68]. Against a chloroquine-resistant *P. berghei* NS strain, 10/10 mice were initially parasite free at both pyronaridine doses, but recrudescence occurred from Day 12 in eight mice in the 25 mg/kg group and from Day 8 in three mice in the 50 mg/kg group; one mouse in each group died. However, it was also noted that amodiaquine (200 or 400 mg/kg/4 days), mefloquine (200 or 400 mg/kg/4 days) and artemisinin (800 mg/kg/4 days) were completely ineffective against this strain [68].

Pyronaridine (SC/4 days) efficacy in mice for seven *P. berghei* lines with resistance to various anti-malarials was similar to the chloroquine-susceptible *P. berghei* N strain [49]. Against chloroquine-resistant *P. berghei* RC, the ED_50_ was 0.5 mg/kg/day, similar to the ED_50_ against *P. berghei* N (0.4 mg/kg/day), whilst the ED_90_ was 10.0 mg/kg/day and 0.9 mg/kg/day, respectively [49]. Only against quinine-resistant *P. berghei* was the pyronaridine ED_50_ increased (to 38 mg/kg/day) [49]. Pyronaridine efficacy was unimpaired against chloroquine-resistant and mefloquine-resistant *P. yoelii versusP. yoelii nigeriensis* (ED_50_ 0.6, 0.9, 0.3 mg/kg/day, ED_90_ 1.2, 1.4. 0.7 mg/kg/day, respectively) but was decreased for a halofantrine-resistant strain (ED_50_ 8.0 mg/kg/day, ED_90_ >100 mg/kg/day) [49].

In mice infected with lethal multi-drug resistant *P. yoelii nigeriensis* (resistant to chloroquine, mefloquine, quinine, amodiaquine, mepacrine and halofantrine), parenteral pyronaridine (> 2.5 mg/kg/day for 3 or 4 days) was highly effective against blood schizonts, protecting animals completely (n = 54), whereas 14/14 control mice died [69]. A similar experiment using the same multidrug-resistant strain found that oral pyronaridine 10 mg/kg/day for seven days (n = 14) protected mice against mortality for 28 days, though there was a transient parasitaemia at Day 24 [70]. However, a 15 mg/kg/day dose (n = 19) for seven days was completely curative. All control animals died by Day 10 (n = 10) [70].

Cross-resistance between pyronaridine-resistant strains and other anti-malarials has also been investigated. When the highly pyronaridine-resistant *P. berghei* PR line was used, as expected, all mice treated with pyronaridine (400 mg/kg/4 days) had parasitaemia at Day 4 and 4/10 mice died; cross resistance was seen with mefloquine, amodiaquine and artemisinin [68]. Further experiments in mice infected with the pyronaridine-resistant *P. berghei* PR line indicated cross resistance with chloroquine, piperaquine, quinine and quinacrine [61]. However, pyrimethamine and sulphadoxine retained activity, and in combination showed synergistic activity against this strain. Artemisinin was also effective, but at a higher dose than for the pyronaridine-susceptible strain [61].

### Effect in combination therapy with artemisinins

The *in vivo* efficacy of pyronaridine has been studied in combination with both artemisinin and artesunate [11,42]. Importantly, there is some evidence to suggest synergy between pyronaridine and artemisinin/artesunate, restoring efficacy against strains resistant to the drugs individually. This may have significant implications for delaying the selection and spread of resistance.

Pyronaridine (subcutaneous/4 days) ED_50_ in mice was 1.4 mg/kg/day against chloroquine-resistant *P. yoelii* NS, 48.5 mg/kg/day against an artemisinin-resistant strain and 17.0 mg/kg/day against a pyronaridine-resistant strain; corresponding values for artemisinin were 12.0, 47.5 and 15.2 mg/kg/day. Interestingly, though the effect of pyronaridine−artemisinin-based combination therapy was only additive against the NS strain, there was marked synergism for both parasites that were resistant to one or both anti-malarials [42].

These findings have recently been confirmed in a study of pyronaridine−artesunate (3:1 ratio) in *P. berghei* infection in mice [11]. Against *P. berghei* NY (drug susceptible), the ED_50_ for pyronaridine was 0.42 mg/kg, artesunate was 5.1 mg/kg and the combination was 1.12 mg/kg and the ED_90_s were 0.8, 31.19 and 1.87 mg/kg, respectively [11]. Against a pyronaridine-resistant strain (NPN) the ED_50/90_ for pyronaridine was 0.57/30.83 mg/kg, for artesunate was 0.24/204.17 mg/kg and the combination 0.42/13.06 mg/kg. Against artesunate-resistant *P. berghei* (SANA) the ED_50/90_s for pyronaridine, artesunate and combination respectively, were 0.89/2.29 mg/kg, 4.50/420.73 mg/kg and 1.46/2.10 mg/kg. Based on ED_90,_ pyronaridine was 38.5-fold less active against the NPN strain *versus* NY, whereas the combination was only seven-fold less active. The combination had almost the same activity against the SANA strain as for the NY strain. Interestingly, the combination was more active against both resistant strains than either pyronaridine or artesunate alone [11].

In a curative model of *Plasmodium chabaudi* infection in mice, pyronaridine 3 mg/kg or artesunate 4 mg/kg were ineffective when given alone over three days [11]. In fact, pyronaridine 6 mg/kg was needed to cure 5/5 mice for 28 days. However, just 3 mg/kg pyronaridine plus 1 mg/kg artesunate was effective in suppressing infection in 4/5 mice for 28 days and in sub-inoculated mice for a further 28 days without recrudescence [11]. A 6 + 2 mg/kg and 12 + 4 mg/kg pyronaridine plus artesunate dose was effective in 5/5 mice throughout the observation period [11]. This study suggests that pyronaridine plus artesunate in combination may allow a lower dose of pyronaridine to be used than when administered as monotherapy.

## Primate models

Pyronaridine has also been tested for schizontocidal activity in primate models of malaria infection. In *Plasmodium inui*-infected rhesus monkeys (n = 3) given a single oral dose of pyronaridine 28 mg/kg, asexual parasitaemia was cleared in 64−144 hours, with recrudescence occurring 11−33 days after dosing [64].

Pyronaridine (10 mg/kg) had no effect on the schizontocidal effect of primaquine against *Plasmodium cynomolgi* sporozoites in rhesus monkeys (n = 3) [59]. Similarly, oral pyronaridine (24 mg/kg/day/6,/3, or/1 days) had no tissue schizonticidal effect in rhesus monkeys infected with *P. cynomolgi*, though a residual blood schizonticidal action was observed [62].

Intragastric pyronaridine did not perform as well as chloroquine in monkeys infected with *P. cynomolgi*[58]. Pyronaridine 6 mg/kg for three days cleared parasitaemia in 2/4 monkeys in a mean time of 77 ± 44 h, though chloroquine cleared parasitaemia in 4/4 monkeys in 66 ± 12 h. A single dose of 30 mg/kg pyronaridine in this model cleared parasitaemia (n = 2) in 54 ± 6 h, similar to the clearance time with chloroquine (n = 2, 60 ± 17 h). A 30 mg/kg dose but given over three days cleared parasitaemia in 66 ± 12 h with pyronaridine (n = 4) and 52 ± 14 h with chloroquine (n = 4). However, all six monkeys treated with pyronaridine 30 mg/kg had recrudescence at 12−22 days, followed by four to five recrudescences on repeated therapy. There was one recrudescence in the 30 mg/kg over three days chloroquine group with no recrudescence after a second treatment course [58].

Intramuscular pyronaridine 9 mg/kg given over two days to *P. cynomolgi*-infected monkeys (n = 4) cleared parasitaemia in 48−120 h with recrudescence at 10−18 days [64]. In the same model (n = 5), higher doses of pyronaridine (IM 20 mg/kg in two divided doses six hours apart) cleared parasitaemia after three to four days, and cured all monkeys with no recrudescence over three months of follow up [71,72].

Intravenous pyronaridine 6 mg/kg over one hour (n = 3) cleared *P. cynomolgi* parasitaemia in 73−97 h with recrudescence at seven to eight days; the same dose of chloroquine (n = 3) cleared parasitaemia in between 93−120 h with recrudescence within one to two days [64]. Intravenous pyronaridine 2−5 mg/kg given to monkeys infected with *Plasmodium knowlesi* (n = 7) cleared parasitaemia within 48−72 h with recrudescence at four to 11 days [64].

## Gametocytocidal activity

Initial *in vitro* data indicated strong gametocytocidal activity of pyronaridine against two multi-drug resistant *P*. *falciparum* isolates (KT1 and KT3) cultured for 14 days after 48 h of drug exposure [63]. Pyronaridine had an IC_50_ of 6 nM for KT1 and 20 nM for KT3 and was more active than four other agents, including primaquine (IC_50_ 800 nM and 2100 nM, respectively).

However, these findings are not supported by *in vivo* tests. After 0.1 mg/kg pyronaridine was given to *P. berghei* ANKA-infected mice, there were no changes in gametocyte density or morphology two or 24 h after administration [59]. When *Anopheles stephensi* were fed on mice infected with *P. berghei* (ANKA) after treatment with pyronaridine (0.5 mg/kg) plus sulphadoxine−pyrimethamine (0.3 mg/kg, 2:1), sulphadoxine−pyrimethamine (0.3 mg/kg) alone, or sulphadoxine (0.2 mg/kg) and pyrimethamine (0.1 mg/kg) separately, there was no impact of pyronaridine on the oocyte gut-positive or gland-positive rates in the dissected mosquitoes [65]. Further, similar experiments confirmed no additional effect of pyronaridine on gametocytes when added to sulphadoxine−pyrimethamine [59].

In terms of gametocydal activity in man, clinical evidence from adults and children shows no relevant effect of pyronaridine monotherapy on gametocyte carriage of * P. falciparum *[73]. However, chloroquine was associated with a higher relative risk (11.5) of post-treatment gametocytaemia *versus* pyronaridine (1.25) in patients who had chloroquine-resistant infection [73]. This suggests a relative benefit of pyronaridine *versus* chloroquine for gametocyte carriage in areas where chloroquine resistance may be a problem.

In patients treated for *P. falciparum* malaria with pyronaridine in combination with sulphadoxine−pyrimethamine (800 + 1000 + 50 mg over two days or 500 + 1500 + 75 mg or 500 + 1000 + 50 mg/1 day) gametocytemia remained high: 47/57 (82.5%) at Day 14 and 41/57 (71.9%) Day on 28, though gametocyte density was reduced from 254.2 parasites per μL on Day 14 to 27.5 parasites per μL on Day 28 [59]. Examination of batches of *Anopheles dirus* that were fed on six gametocytaemic patients four, eight, 10, 14, 19 and 27 days after treatment showed that although many oocysts were retarded, sporogony of *P. falciparum* was not completely suppressed [59].

There is one report including 12 patients with initial *Plasmodium malariae* and *P. ovale* gametocytes who had complete gametocyte suppression at Day 3 after pyronaridine therapy [74]. However, overall, pyronaridine should be considered to have no clinically relevant effect on *P. falciparum* gametocyte carriage.

## Toxicological profile

Single oral dose toxicology studies in the rat demonstrated that the inherent acute toxicity of pyronaridine was low with findings of decreased body weight gain, diarrhoea and soft stools at ≥1,000 mg/kg and chromaturia at 2,000 mg/kg.

The effect of 3 days of oral dosing with pyronaridine was examined in the dog and primate. In dogs doses up to 240 mg/kg pyronaridine (total dose) resulted in vomiting, but no other serious adverse effects. Hyperaemia of the intestinal mucosa and focal hyperaemia of the gastric mucosa was noted in 1 animal [64,72]. In monkeys, pyronaridine (240 mg/kg total dose) elevated serum glutamate pyruvate transaminase (SGPT) in one animal returning to normal after one week, there were no other adverse effects [64,72].

Intramuscular pyronaridine was examined in mice (LD50 was ~250 mg/kg), rabbits (20, 40 and 80 mg/kg; one death occurred at 80 mg/kg and ECG changes at all doses with the number of changes increasing with dose), and dogs (20, 40 and 60 mg/kg; one death occurred at 60 mg/kg, convulsions in another animal and elevated SGPT in a third) [64,72].

In recent sub-acute toxicity studies in the rat and dog the NOAEL following 28 days of treatment was 23 mg/kg/day and 5 mg/kg/day, respectively. Findings included yellow colouration of skin, eyes, urine and tissues/organs, accumulation of basophilic material usually accompanied by chronic inflammation predominantly in liver, bone marrow, spleen, lung and kidney. Marked hepatocellular hypertrophy and hyperplasia in spleen and bone marrow were also observed in the rat and decreased red blood cell parameters and increased liver enzymes were observed in the dog. Partial or total recovery was evident in some tissues after a two-week recovery period but in other tissues an increase in severity was noted [75].

Earlier repeat dose toxicology studies had been conducted in rats and dogs. In rats were given 40 or 200 mg/kg/day oral pyronaridine for 14 days. All animals had retarded growth and three died within the 14-day study period in the 200 mg/kg/day group. [64,72]. In dogs, oral pyronaridine 12 or 24 mg/kg/day for 30 days was well tolerated with no changes in laboratory parameters or ECG. [5,64,72]. In rabbits, IV pyronaridine (20 mg/kg on Day 1 and 10 mg/kg for Days 2−7) showed good tolerability [64,72].

Pyronaridine in combination with artesunate 28-day oral dosing in rats, and cyclic dosing studies in the rat and dog (three consecutive days dosing per week for two or four weeks) showed that the findings were in line with those observed for pyronaridine alone. Results from the 4-cycle studies showed extensive tissue accumulation of pyronaridine and, although the 6 (rat) or 8 (dog) week recovery study was sufficiently long for some of the histopathological changes to have resolved, other changes, such as inflammatory and degenerative changes in liver and the presence of basophilic granules and macrophage accumulation in various tissues, persisted [76].

Pyronaridine possesses some potential for *in vitro* genotoxicity as shown in the bacterial mutagenicity test, due to its property as an intercalator. Its “genotoxic” effects in mammalian cells however, i.e. the induction of chromosomal aberrations and, in particular, polyploidy, are considered related to its property as a catalytic inhibitor of topoisomerase II [76]. A number of studies were performed to examine the *in vivo* genetic toxicity profile of pyronaridine including micronucleus studies, a liver UDS study and a COMET assay. These studies provided uniformly negative results at the highest tolerated doses of pyronaridine [76].

In reproductive toxicity studies pyronaridine had no effects on rat male fertility at doses up to 180 mg/kg/day. Pyronaridine tetraphosphate when dosed daily during the sensitive periods of embryonic organogenesis to pregnant rats and rabbits) at levels up to 420 and 120 mg/kg/day, respectively resulted in maternal toxicity at the highest levels but no evidence of teratogenicity [76].

Othe potential toxicities of pyronaridine were examined. There was no evidence of cytotoxicity [8,77] or phototoxicity [78]. A mild local injection site irritation was noted in rabbits dosed IM [5]. Transient increases in serum alanine aminotransferase (ALT) were reported in 1 monkey and 1 dog, but no consistent increases were observed in animal studies.

A series of safety pharmacology studies were undertaken with pyronaridine. In brief these studies demonstrated that pyronaridine decreased urine volume with a concomitant increase in density and electrolyte concentration at 500 mg/kg, decreased gastric acidity and volume of secretion at doses of 100 mg/kg and above in the rat. Decreases in body temperature were noted in the mouse (300 and 1000 mg/kg, but not the dog (up to 60 mg/kg). Pyronaridine produced a significant analgesia in the acetic acid writhing test (≥ 300 mg/kg). No effects were noted in the following studies: Irwin, spontaneous motility, motor co-ordination, hexobarbital sleep time, hot-plate test, convulsion induction, isolated ileum, GI transit time, respiratory rate and tidal volume, and cardiovascular parameters in the telemetered dog. *In vitro* cardiovascular studies (hERG test and Purkinje fibre assay) showed that pyronaridine inhibited the hERG channel with an IC_50_ of 0.65 μM. In the Purkinje fibre study at a concentration of 3,500 ng/mL small decreases in action potential duration and the rate of rise of the action potential upstroke were observed, but not at lower concentrations, indicating that pyronaridine blocks sodium, calcium and the hERG potassium channel.

The toxicological profile of oral pyronaridine was initially investigated at the Institute of Parasitic Diseases in the Chinese Academy of Medical Sciences, and published, in Chinese, in their annual reports [60,64,79]. These data have also been reviewed in English by Shao, Fu and Xiao, Chen and Zheng, and Chang *et al.*[3,5,72,80]. The main findings reported in these papers have been collated and summarized below, with reference to any additional data. Overall, the acute and sub-acute toxicity of pyronaridine was generally less than that of chloroquine in all animal species tested (Additional files 3 and 4) [5,63,66,71,79,80]. Cardiovascular toxicity was also less than that of chloroquine [3,5,71,79]. There were no unexpected findings that would be a particular cause for concern at therapeutic levels in human subjects. However, evidence of embryotoxicity in rodents suggests that pyronaridine should be used with caution during pregnancy [76,81].

## Acute toxicity

The main findings from acute toxicity studies with pyronaridine are summarized in Additional file 3[5,63,66,71,79,80].

### Oral administration

LD_50s_ for oral pyronaridine have been reported in a number of studies [63,66,71,80] and these are referred to in Additional file 3. Dogs given oral pyronaridine 120 mg/kg (n = 2) or 240 mg/kg (n = 2) (25% of total dose on Day 1 then once daily for the next two days) experienced vomiting, but no other serious adverse effects. Autopsy was carried out in two dogs, one of which had hyperaemia of the intestinal mucosa and focal hyperaemia of the gastric mucosa [63,71].

In monkeys (n = 4), pyronaridine 240 mg/kg (60 mg/kg twice on Day 1 then once daily for two days) elevated serum glutamate pyruvate transaminase (SGPT) in one animal from 20 to 107 IU/L, returning to normal after one week, though there were no other adverse effects [63,71].

In a recent study there were no mortalities up to and including a dose of 2,000 mg/kg in the rat. Clinical findings of diarrhoea and soft stools were found at ≥1,000 mg/kg and one incidence of chromaturia was reported at 2,000 mg/kg in males. Suppression of body weight gain was observed from Day 1 to Day 3 of the study in 1,000 and 2,000 mg/kg groups [82].

### Non-oral administration

IM pyronaridine LD_50_ in mice was reported as 250.6 ± 33.1 mg/kg, 2.8-fold higher than that of chloroquine (89.7 ± 34.0 mg/kg) [63,71].

In rabbits, a single IM dose of pyronaridine 80 mg/kg (n = 5), 40 mg/kg (n = 4) or 20 mg/kg (n = 5) resulted in one death after 40 min (80 mg/kg, minimal lethal dose [MLD]). Electrocardiogram (ECG) changes, including premature ventricular beats and ventricular fibrillation were seen at the 80 mg/kg dose. Reversible bradycardia was observed at the 40 mg/kg dose, with prolonged QRS, P−R and Q−T, and arrhythmia. Prolonged QRS was seen at the 20 mg/kg dose, resolving within one hour after dosing as well as arrhythmia. Otherwise, pyronaridine was well tolerated. In comparison, mortality was 3/3 with chloroquine 80 mg/kg, 1/5 at 40 mg/kg and 1/4 at 20 mg/kg within 24 h after administration; reversible bradycardia was observed with chloroquine at the 20 mg/kg dose [63,71].

In dogs, a single IM dose of pyronaridine 20 mg/kg (n = 5) or 40 mg/kg (n = 5) was well tolerated. Pyronaridine MLD in dogs was 60 mg/kg dose, resulting in 1/3 deaths at 23 min after administration. A clonic convulsion at 20 min and vomiting after 24 min were observed in a second dog, with recovery by the next day, and a third dog had raised SGPT (22.5 to 85 IU/L), resolving within a week. No further adverse effects were observed in surviving dogs over one month of follow up. Comparative data for IM chloroquine showed mortality rates of 2/2 for 20 mg/kg and 1/3 for 10 mg/kg within 24 min. Two surviving dogs receiving 10 mg/kg chloroquine and three given 5 mg/kg showed tremor and white foam spitting, but all recovered within 24 h. ECGs showed delayed conduction with chloroquine, resolving within two hours [63,71].

Intravenous 30 mg/kg pyronaridine given over one hour in rabbits (n = 10) was sub-lethal, though ECG changes were observed, notably fused T and P waves and prolonged P−R and QRS. In this experiment, chloroquine 5 mg/kg caused one rabbit to die from severe hypotension and ECGs showed occasional prolongation of P−R, broadened QRS and bradycardia [3].

## Sub-acute toxicity

The main findings for sub-acute toxicity studies with pyronaridine are summarized in Additional file 4[5,63,71]. Sub-acute toxicity studies were conducted in rats given either 40 mg/kg/day (n = 15) or 200 mg/kg/day (n = 15) oral pyronaridine for 14 days. In the 40 mg/kg/day group, one rat died on Day 13, though the growth and development of the survivors was unaffected. However, in the 200 mg/kg/day group, all animals had retarded growth and three died within the 14-day study period. In comparison, rats given 100 mg/kg/day chloroquine experienced more severe growth retardation than with pyronaridine and three animals died [63,71].

In dogs, oral pyronaridine 12 mg/kg/day (n = 2) or 24 mg/kg/day (n = 2) for 30 days was well tolerated with no changes in laboratory parameters or ECG. The same daily dose of oral chloroquine elicited multiple symptoms including salivating, tremor, vomiting and white foam spitting and all the animals died; death on Day 8 and Day 30 for the 12 mg/kg/day dose (n = 2) and on Day 5 and Day 14 for the 24 mg/kg/day dose (n = 2) [5,63,71].

A sub-acute toxicity study in rabbits with IV pyronaridine 20 mg/kg (n = 5) on Day 1 and 10 mg/kg for Days 2−7 showed good tolerability, though chloroquine (n = 5) at the same dose led to one death after 20 min during the fifth administration [63,71].

In recent sub-acute toxicity studies in the rat the NOAEL following 28 days of treatment was 23 mg/kg/day. Findings included yellow colouration of skin, eyes, urine and tissues/organs, accumulation of basophilic material usually accompanied by chronic inflammation predominantly in liver, bone marrow, spleen, lung and kidney. Marked hepatocellular hypertrophy and hyperplasia in spleen and bone marrow were also observed. Partial or total recovery was evident in some tissues after a two-week recovery period but in other tissues an increase in severity was noted [82].

In the dog the NOAEL following 28 days treatment was 5 mg/kg/day [82]. Findings included loss of appetite, vomiting, body weight loss, decreased red blood cell parameters, increased liver enzymes, increase in liver, lung, spleen, brain and kidney weight, yellow discolouration of many tissues and organs, accumulation of basophilic material associated with chronic inflammation and hyperplasia of a number of tissues.

## Acute and sub-acute toxicity in combination therapy

The LD_50_ in mice or rats was not affected when oral pyronaridine was combined with sulphadoxine−pyrimethamine (100/5 mg/kg) [66]. However, when pyronaridine plus sulphadoxine−pyrimethamine at 50%, 25% or 12.5% of the LD_50_ were tested there was an additive toxic effect [66]. In mice, oral pyronaridine 600 mg/kg had a slight antagonistic effect on the toxicity of primaquine, marginally increasing LD_50_ by about 34%; lower doses had little impact on primaquine toxicity [80]. Pyronaridine also had a protective effect on murine seven-day mortality for primaquine 50 mg/kg given one hour after pyronaridine dosing: 12/30 mice died with primaquine alone *versus* 6/30 that had received pyronaridine 507 mg/kg (0.38 LD_50_) (*P* < 0.05) [80]. A similar experiment in rats given IM pyronaridine plus oral primaquine (51 mg/kg) one hour later showed no effect of the combination on seven-day mortality *versus* primaquine alone [80]. The LD_50_ in rats was not affected when oral pyronaridine was combined with artesunate (pyronaridine tetraphosphate: artesunate (3:1)), with similar findings to those for pyronaridine alone. In addition a decrease in locomotor activity was observed.

The sub-acute toxicity of pyronaridine combined with artesunate was studied in a 28-day study in rats, and cyclic dosing studies in the rat and dog (three consecutive days dosing per week for two or four weeks). In these studies the findings were in line with those observed for pyronaridine alone. In addition, in the rat the following changes were observed: increased haematopoiesis in the spleen, thymic atrophy, hepatocyte hypertrophy which was considered to be an adaptive change due to enzyme induction in the liver, reactive hyperplasia of the lymph nodes, follicular epithelial hypertrophy of the thyroid glands and perivascular basophilic vacuoles with associated inflammation. A number of these changes recovered during a six-week recovery period, however, the changes in the liver, kidney and spleen as well as the thymic atrophy persisted [82]. As for the rat, in the dog the findings were in line with those observed for pyronaridine alone [82]. In addition, increased haematopoiesis in the spleen and perivasculitis in the brain were observed. After an eight-week recovery period most changes had either resolved or were greatly reduced, however, the changes in the liver, gall bladder and kidney, along with adaptive changes in the spleen and bone marrow persisted.

## Cardiovascular toxicity, intravenous challenge

In anaesthetized rabbits (n = 10), 4 mg of pyronaridine (2 ml in 2% solution) was injected every two minutes through the carotid vein. A 40 mmHg blood pressure drop was obtained with a mean pyronaridine dose of 39.8 ± 9.6 mg/kg *versus* 11.4 ± 3.1 mg/kg for chloroquine (*P* < 0.01) [63,71]. ECG changes observed with pyronaridine included fused T and P, prolongation of the QSR, P−R and Q−T intervals and broadened S, followed by A−V block, sinus bradycardia, premature ventricular contraction leading to auto-ventricular rhythm and death. The mean lethal dose in this study was 64.6 ± 10.1 mg/kg for pyronaridine *versus* 20.7 ± 3.8 mg/kg for chloroquine (*P* < 0.01) [63,71].

A comparable experiment conducted in dogs (n = 5) given 10 mg pyronaridine (5 ml every two minutes) indicated hypotension at a dose of 97.8 ± 18.7 mg/kg *versus* 28.8 ± 4.4 mg/kg for chloroquine (*P* < 0.01). ECG changes with pyronaridine in dogs were similar to those observed in rabbits. The lethal pyronaridine dose in dogs was 116.0 ± 22.3 mg/kg *versus* 47.4 ± 6.7 mg/kg for chloroquine [63,71]. A cross-over study in anaesthetized cats (n = 3) compared the effect on blood pressure of pyronaridine 2 mg/kg and 4 mg/kg with that of chloroquine at the same dose. The decrease in blood pressure with pyronaridine 2 mg/kg and 4 mg/kg was 6 and 20 mmHg at Day 1 and 20 and 40 mmHg on Day 2, respectively. Corresponding results for chloroquine 2 mg/kg and 4 mg/kg were 20 and 35 mmHg and 35 and 68 mmHg, respectively. Thus, chloroquine was between 1.7- and 3-fold more toxic than pyronaridine [5].

## Mutagenicity/embryotoxicity

Pyronaridine mutagenicity was studied in the *Salmonella* microsome system [83]. Five strains of histidine-requiring *Salmonella typhimurium* (TA100, TA98, TA1535, TA1537 and TA1538) with and without S-9 were used. Positive controls included hycanthone and furapromide. Pyronaridine did not induce mutations in four strains, but there was evidence of a mutagenic effect without S-9 for strain TA1537 that was dose-dependent between 100−1,000 μg/plate [83]. There was no structural damage to chromosomes or the spindle apparatus with pyronaridine at the LD_50_ in the mouse micronucleus test [84]. There was no effect of pyronaridine at 0.78 μg/ml on chromosomal aberration in Chinese hamster fibroblast cells, though aberration rates were 5−9.9% at the highest dose tested (2.34 μg/ml, 50% of growth inhibiting dose) [5,85]. Mice were treated with 60, 685, 1370 mg/kg pyronaridine and bone marrow smears were carried out 24 h after treatment. No mutagenic activity was found [85].

A comprehensive battery of *in vitro* and *in vivo* genetic toxicity studies has been conducted. In the Ames test, no evidence of bacterial mutagenicity was observed, and a COMET assay showed no evidence for primary DNA damage. An increased frequency of chromosomal aberrations in Chinese hamster lung and evidence of genetic toxicity was observed in the mouse lymphoma assay (±S9) [85]. *In vivo,* a series of mouse micronucleus assays with pyronaridine were undertaken. Two assays were reported as negative and two reported as positive. It is noteworthy that both of the ‘positive’ studies with pyronaridine utilized a bone marrow staining procedure (May Grunwald/Giemsa stain) that would be unable to discriminate between’true DNA-containing micronuclei’, and potential artefact arising from non-DNA containing granules, such as the RNA-containing granules (‘Q-bodies’) such as has reported in mouse bone marrow after treatment with quinacrine.

Maier and Schmid reported that quinacrine hydrochloride induced, not micronuclei, but irregularly shaped granular and fibrillar bodies that were negative in the Feulgen reaction [86]. It has also been shown that the use of more specific stains such as acridine orange allow the discrimination of such bodies from true micronuclei [87]. Such fibrillar bodies, although induced by the treatment with quinacrine and thus observed in a dose-dependent frequency, are not due to chromosome breakage, and hence not indicative of any *in vivo* genotoxicity for quinacrine. It is noteworthy that pyronaridine possesses a close structural resemblance to quinacrine. In order to clarify the situation, an additional mouse bone marrow micronucleus test was performed using the same strain of mouse as in the reported ‘positive’ studies (male ICR mice), and in which the smears were prepared, and stained with a DNA specific stain (acridine orange) prior to micronucleus analysis [87]. The results indicated that there was no increase in micronucleus frequency in the bone marrow of male mice dosed with pyronaridine (750 mg/kg). It was concluded that the apparent increases in micronucleus frequency reported in the earlier studies on pyronaridine were due to the presence of non-DNA containing artefacts on the slides, which had been incorrectly identified as ‘true micronuclei’ as a consequence of the use of a staining procedure that was not specific for DNA. Furthermore, an *in vivo* COMET assay examining the effects of pyronaridine in the liver and an unscheduled DNA synthesis assay provided no evidence for a direct DNA damaging effect of pyronaridine and were both negative.

In order to explain the discrepancy between the *in vitro* and *in vivo* data the effects of pyronaridine on topoisomerase II were examined in an inhibition assay and a TARDIS (Trapped in Agarose DNA Immunostaining) study [82]. It was shown that pyronaridine is a weak topoisomerase II catalytic inhibitor with an IC_50_ of approximately 32.7 μM. At *in vitro* concentrations below 5–8 μM topoisomerase II activity was not affected. In these studies there was no evidence that pyronaridine was acting as a topoisomerase II poison.

In addition, the effect of pyronaridine on topoisomerase II in mammalian cells was investigated using the TARDIS assay. The results indicated that pyronaridine was increasing the number of topoisomerase II molecules complexed with cellular DNA, and that it thus may be acting as a topoisomerase II poison. This could be due to a direct interaction between pyronaridine and topoisomerase II or due to an indirect mechanism such as inhibition of removal/repair of the topoisomerase II complexes on DNA. Clearly this conclusion contrasts with that discussed for the previous study in which purified human topoisomerase II enzyme was used and the absence of linear DNA indicated that pyronaridine was acting as a catalytic inhibitor rather than as a topoisomerase II poison. However ‘stable cleavage complex’ formation was seen over a higher test concentration range than that used in the “catalytic inhibition” study [82].

The current data on pyronaridine indicates that the compound may act as both a catalytic inhibitor and a topoisomerase II poison dependent upon exposure concentration. It is clear that either mechanism of action would explain the *in vitro* clastogenicity of pyronaridine towards Chinese Hamster CHL cells and the induction of small colony *tk* mutants in mouse lymphoma L5178Y cells. In male mouse dominant lethal tests there was no evidence of a mutagenic effect or any effect on gametogenesis or fertilization with pyronaridine [88]. In this study, groups of 10 male mice were treated with oral pyronaridine 1,029, 514.5 or 258 mg/kg over three days before mating; one male in the highest dose group died. Mated females were sacrificed on Day 15 after mating. *Versus* controls there were no apparent differences for the pyronaridine-treated group in the percentage of females with implants, average implants, average live foetuses and foetus death rate [88].

Embryotoxicity, but no teratogenicity has been observed in rats with pyronaridine [80,81]. In a study by Ni *et al.*, oral pyronaridine at 84 mg/kg (n = 20), 165 mg/kg (n = 19) or 330 mg/kg (n = 19) for three days or 1,100 mg/kg (n = 19) for one day beginning from D7 of gestation increased the foetal resorption rate in rats in a dose-dependent manner [76]. Pyronaridine also delayed ossification of the occipital bone and sternum of foetuses, though no visceral or skeletal abnormalities were observed [76]. Shao *et al.* administered pyronaridine 10 mg/kg (n = 14) or 20 mg/kg (n = 10) to male rats for 60 days before mating and female rats from 14 days before and throughout gestation [81]. The rate of resorbed foetuses was 13.3% (4/30) for 10 mg/kg and 29.5% (13/44) for 20 mg/kg pyronaridine, the latter being significantly higher *versus* controls (7.5% [11/146], *P* < 0.05). Foetal mortality was also increased for the 20 mg/kg dose *versus* controls: 29.5% (13/44) *versus* 14.4% (21/146), *P* < 0.05. The number of live litters was 2.3 ± 2.7 in the 20 mg/kg pyronaridine group, significantly lower than for the control group (5.7 ± 2.8, *P* < 0.01). No evidence of embryotoxicity was observed in the F_1_ generation. No external or skeletal abnormalities were observed in the F_1_ and F_2_ generation rats [81].

In a recent series of studies pyronaridine did not adversely affect male reproductive function. The NOAEL was considered to be >180 mg/kg/day for reproductive performance and early embryonic development for parental animals. In rats, the NOAEL was between 6 and 47 mg/kg/day for dams. For embryo-foetal development, the NOAEL was 140 mg/kg/day in rats and 40 mg/kg/day in rabbits. Pyronaridine at 420 mg/kg/day resulted in a decrease in foetal weight in rats. At 120 mg/kg/day in rabbits, mean foetal weight reduction and a reduction in the number of ossification centres of the first and second phalanges in both hind limbs was observed. These effects are considered to be a consequence of the maternal toxicity observed at these doses. The NOAEL for pyronaridine are considered to be 30 mg/kg for F1 offspring and >150 mg/kg for F2 foetuses [82].

## Other toxicity

### Cytotoxicity (neutrophils)

At one point amodiaquine was withdrawn from clinical use after reports of neutrophil toxicity resulting in fatal agranulocytosis [89]. As a consequence, it was considered important to re-evaluate the possibility of similar adverse events occurring with both new anti-malarials and those in current use. Pyronaridine is accumulated in lysosomes, but no more so than with other anti-malarials [89]. Moreover, there was no evidence of glutathione depletion with pyronaridine, no significant toxicity to polymorphonuclear leukocytes or inhibition of their function [89]. Oxidation of pyronaridine to a quinoneimine metabolite has been shown to deplete neutrophil glutathione *in vitro*[77]. However, *in vivo* studies in rats failed to find any quinoneimine metabolites in bile samples after pyronaridine dosing [8,90]. The clinical relevance of these findings is unknown, and to date there is no evidence of significant neutrophil toxicity associated with pyronaridine use in humans.

### Phototoxicity

No photoxic reactions were noted in mice exposed to ultraviolet radiation for 24 h after IG administration of pyronaridine 600 mg/kg (n = 20) or 800 mg/kg (n = 18). Slight phototoxicity was seen with chloroquine 300 mg/kg (n = 9) and 600 mg/kg (n = 10) in this study (swelling and auricular erythema)[78]. In another study, no phototoxicity was seen in mice given IG pyronaridine 300 mg/kg (n = 10) or chloroquine 100 mg/kg (n = 10) following irradiation with black light for 24 h [91].

### Injection site reaction

In rabbits, IM pyronaridine 40 mg/kg in 4% solution caused mild local injection-site irritation that resolved within two weeks [5].

### Hepatotoxicity

Isolated and transient elevation of serum alanine aminotransferase (ALT) was reported after acute dosing of pyronaridine in one monkey (from 20 to 107 IU/L, returning to normal after one week) with no concomitant adverse effects. A transient rise in ALT (22.5 to 85 IU/L), which resolved within a week, was reported in a dog following a 60 mg/kg single dose [63,71]. Transient increases in concentrations of hepatic enzymes have been reported during the clinical programme of Pyramax (pyronaridine-artesunate). In a few subjects, aspartate aminotransferase (AST) and (ALT) concentrations of three or more times the upper limit of normal were reported, with a concomitant increase in total bilirubin concentrations that were two or more times the upper limit of normal, although there was no increase in alkaline phosphatase concentrations.

## Safety pharmacology

In a battery of safety pharmacology studies, the cardiovascular, neurobehavioural, motor coordination and respiratory effects of pyronaridine tetraphosphate were examined. Additional studies were undertaken examining the effects on analgesia and the GI and renal systems.

Pyronaridine inhibited hERG [human ether-à-go-go related gene] tail current with an IC_50_ of 0.65 - 0.82 μM. In the rat Langendorff significant decreases in LVP, LVDP, HR, DP and CFR were observed at 10 μM pyronaridine. In a dog Purkinje fibre study, no effect was noted on action potential duration at 350 ng/mL, and a shortening of action potential duration observed at 3,500 ng/mL. *In vivo*, no effects were observed in the dog telemetry study [5,63,71].

No effects were observed on the central nervous system or on pain threshold at doses up to 1,000 mg/kg. A significant analgesia was noted in the acetic acid writhing test at doses of ≥300 mg/kg. No effects were noted using the hot-plate method. Decreases in body temperature were noted in the mouse (≥300 mg/kg), but not in the dog (doses up to 60 mg/kg). A transient increase in respiratory rate was observed at a dose of 500 mg/kg 2 h post-dose. No effect was noted on gut motility, however small, but significant decreases, in gastric acidity and volume of secretion were observed at doses of 100 mg/kg and above in the rat. Significant decreases in urine volume accompanied by increases in density and electrolyte (sodium) concentration were observed with 500 mg/kg pyronaridine [76].

## Pharmacokinetic profile

The pharmacokinetics of pyronaridine have been examined in the rat, rabbit, dog and rhesus monkey. In the rat and dog following intravenous administration of pyronaridine, the blood concentrations of pyronaridine declined in a multi-exponential manner with an apparent terminal half-life of 2 to 4 days. Intramuscular pyronaridine in the rabbit and rhesus monkey reached Tmax within 0.75-1.5 h post-dose with an apparent half-life of 2 to 3 days [92,93].

The metabolism, distribution and elimination of pyronaridine in *in vitro* and *in vivo* models were examined. In brief *in vitro* metabolism showed evidence of 8–12 metabolites. Subsequent profiling showed that all human in citro metabolites were present in rat and dog systems. Following oral administration of pyronaridine no significant metabolism was observed with the major dose related compenent found to be unchanged pyronaridine, although in the rat after IP administration metabolites were observed in urine and faeces. Incubations with recombinant human CYP450 isoforms indicated that pyronaridine could be metabolised by CYP1A2, CYP2D6 and CYP3A4 [76].

Pyronaridine preferentially associates with blood cells and is highly plasma protein bound. In mice, rats and rabbits the highest concentrations of radioactivity were typically located in the liver, spleen, adrenal gland, kidney and thyroid. In the rat the half life of elimination was 137–231 h [76].

Examination of the elimination of pyronaridine in the rat (60 mg/kg dosed) found that around 93% wsas excreted over 14 days, with the majority in the faeces. In the dog (dose of 9 mg/kg) an overall recovery of 44% was noted over the same duration. The shortfall in recovery was investigated and a significant proportion of the dose remained associated with the liver [76]. At 10 mg/kg pyronaridine in the rat the majority of the dose was eliminated in the urine [94].

Investigations into the pharmacokinetic characteristics of pyronaridine were held back by the need to develop a sensitive, simple and reliable assay. Sensitive HPLC [high performance liquid chromatography] methods were developed using the anti-malarials amodiaquine or quinine as internal standards [4,95]. Most recently, Naik *et al.* developed liquid chromatography-mass spectrometry (LC-MS) assays for pyronaridine in both urine and blood [92,96]. Both of these techniques used amodiaquine as an internal standard, were reproducible and accurate and had a lower limit of quantitation for pyronaridine of 14.3 ng/ml in urine and 5.7 ng/ml in blood [92,96].

## Pharmacokinetics in animal models

The pharmacokinetics of pyronaridine in rabbit blood was determined using a spectrofluorometric method for IG, IM or IV administration [93]. Intragastric pyronaridine pharmacokinetics were described using a linear open two-compartment model. Mean pharmacokinetics for 30 mg/kg (n = 3) or 60 mg/kg (n = 3) were, respectively: T_1/2_ 56.0 ± 7.0 h, 56.0 ± 7.0 h; Cmax 768 ± 17.0 ng/ml, 1,514 ± 376 ng/ml; and Tmax 1.38 ± 0.22 h, 1.62 ± 0.37. Using the same model as for intragastric administration, intramuscular pyronaridine (6 mg/kg) pharmacokinetics showed complete and rapid absorption with a k_a_ of 33.54 ± 21.81 h^-1^ and a Tmax of 0.75 ± 0.44 h. Intragastric pyronaridine was 34.6% bioavailable compared with IM administration, with a k_a_ of 2.4 ± 1.26 h^-1^[93]. A further study in rabbits of 20 mg/kg IM pyronaridine had a comparable T_1/2_ of 49 h and Cmax was reached within one hour after dosing [4]. Using a linear three-compartment open model, mean (± SD) pharmacokinetic parameters after pyronaridine 6 mg/kg IV bolus (n = 4) were: T_1/2_ 59.0 ± 10.0 h; V_c_ 2.418 ± 0.287 L/kg; V_d(ss)_ 29.0 ± 6.0 L/kg; CL_T_ 0.442 ± 0.131 L/kg.h.

Pyronaridine blood concentrations in rhesus monkey (n = 1) after oral administration of 160 mg/week for three weeks were sampled at 0.5 h, then hourly for eight hours then at Days 1, 2, 3, 7, 14, 21 and 28 [97] Pyronaridine blood concentrations after three weeks of 160 mg/week were undetectable on Weeks 2 and 3. With a 540 mg/week oral dose, pyronaridine concentrations at Week 3 were 88 ng/ml four hours after dosing and 142 ng/ml at 30 h [97]. With IM administration of 160 mg/kg for three weeks in the same model, the highest concentration of pyronaridine was recorded one hour after the Week 3 dose (1983 ng/ml) [97]. Pyronaridine concentrations had declined below the limit of detection by 21 days; the T_1/2_ was 64 h [97]. Pyronaridine concentrations in urine for the 24-h period after Week 2 dosing after IM administration were approximately 20-fold higher than with oral dosing [97].

The pharmacokinetics of pyronaridine have been investigated following single intravenous and oral administration to the rat and dog [76]. Following intravenous administration of pyronaridine to rats and dogs, the blood concentrations of pyronaridine declined in a multi-exponential manner with an apparent terminal half-life of 2 to 4 days. The total blood clearance was low, at less than 30% hepatic blood flow, and the volume of distribution was high, indicating extensive distribution to tissues. Oral bioavailability was calculated to be 42% in the rat and 35% in the dog. Co-administration of pyronaridine with artesunate (both 10 mg/kg) in the dog reduced the exposure to pyronaridine by approximately 2-fold following both oral and intravenous administration.

### Metabolism

In a rhesus monkey given oral or IM pyronaridine 160 mg/week for three weeks, no metabolites were seen in whole blood, though evidence for at least one metabolite was found in urine at a concentration of 100−200 ng/ml in samples collected seven days after dosing [97]. In rats (n = 4), no glutathione conjugates or quinoneimine metabolites could be detected in bile samples from animals given pyronaridine [8].

More recently, *in vitro* studies in which pyronaridine was incubated with rat liver microsomes generated 11 different metabolites, whereas incubation with human liver microsomes generated nine metabolites [90]. Six metabolites were observed in both rat and human microsomes, five were only seen in rat and three only in human microsomes [90]. Subsequent metabolite profiling with ^14^ C] pyronaridine in rat, dog and human liver microsomes showed all human *in vitro* metabolites to be present in both rat and dog systems, however no structural identification was attempted because of the low metabolic turnover [76]. *In vivo* studies in rats given intraperitoneal (IP) pyronaridine 50 mg/kg found 14 different metabolites in urine and faeces. Unchanged pyronaridine was excreted mainly in the urine and metabolites in the faeces. Three metabolites were observed only in faeces. Overall, across the *in vitro* and *in vivo* studies, there were three main pathways for pyronaridine metabolism: i) aminoquinoline conversion to quinoneimine, ii) pyrrolidine ring hydroxylation or carbonylation, and iii) *O*-demethylation. Quinoneimine metabolites of aminoquinolines are of interest as they are thought to cause the toxicity problems seen with amodiaquine [98]. However, quinoneimine metabolites were detected only in the microsome studies, not in urine or bile from *in vivo* studies. This suggests that, either the other available metabolic pathways are preferred, or that these metabolites are easily reduced *in vivo*. *O*-demethylation was detected in rat, but not human microsomes [90].

Following oral administration of ^14^ C] pyronaridine to rat and dog, profiling of plasma, urine, faecal and liver extracts indicated a single major dose-related component, which was confirmed to be unchanged pyronaridine. ^14^ C] pyronaridine was the only component in any sample that represented >5% of the dose administered, thus indicating that pyronaridine undergoes no significant metabolism in rat and dog [76]. Incubations with recombinant human CYP450 isoforms indicated that pyronaridine could be metabolised by CYP1A2, CYP2D6 and CYP3A4 [76].

### Distribution and elimination

*In vivo* data from rabbits indicate that pyronaridine is concentrated in blood cells with a blood:plasma ratio ranging from 4.9 to 17.8 with 20 mg/kg IM dosing [4], and between 3−6 during 1−96 h after 6 mg/kg IM dosing [93]. *In vitro* studies using whole blood, indicated that pyronaridine preferentially associates with blood cells with a blood:plasma distribution of 2.1 to 2.4 in rat, 2.5 to 3.8 in rabbit, 2.0 to 2.4 in dog and 1.2 to 1.7 in human. Plasma protein binding of pyronaridine in rat, rabbit, dog and human was high (92-96%) and similar in all species [76]. Pharmacokinetic studies using plasma may, therefore, underestimate pyronaridine concentrations.

The major sites of pyronaridine distribution in tissues 24 h after IG administration of ^3^ H-pyronaridine 30 mg/kg to mice were: liver (17.9%), large intestine and content (1.7%), kidney (1.4%), lung (1.1%), spleen (0.5%) and stomach and content (0.3%); 0.5% was present in whole blood and 0.3% in plasma [99]. Recovery from urine and faeces was 54.3% at 24 h after dosing, and 12.0% remained in the carcass [99]. For IP administration of 30 mg/kg pyronaridine, 20.3% of the dose was present in the liver, 3.3% the large intestine, 1.3% kidney, 0.7% lung, 1.2% in spleen and 0.6% in stomach; 0.4% was present in whole blood and 0.1% in plasma. Residual carcasses contained 20.0% of the dose and 39.6% was excreted in urine and feces. During 12 days after IG and IP administration, the total urinary excretion was 20.83% and 24.07%, and the total faecal excretion was 40.44% and 41.24%, respectively [99].

Quantitative whole body phosphor imaging, following oral administration of ^14^ C-pyronaridine to non-pigmented and pigmented rats showed concentrations of radioactivity were greater than 10-fold higher in tissues than in blood. The highest concentrations of radioactivity were achieved in the liver, spleen, adrenal gland, kidney and thyroid gland, with half-lives of elimination between 137 and 231 h. There was evidence of melanin binding in the eye [76].

In rabbits, one hour following IM 2 mg/kg pyronaridine, the highest drug concentrations were found in lung, spleen and kidney (28.6% of dose); peripheral blood had 2.5% and total recovery was 31.1% [94]. Pyronaridine concentrations had significantly decreased in all tissues 72 h after administration; the highest concentrations were in the spleen, kidney, lung, liver and heart (5.2% of dose); 0.07% was found in peripheral blood [94].

The pharmacokinetics of ^14^ C pyronaridine tetraphosphate was studied in male and female Sprague–Dawley rats. Following oral administration of a single dose (10 mg/Kg) of ^14^ C- pyronaridine tetraphosphate [100], the drug was rapidly adsorbed mainly from the small intestine and it was rapidly distributed as evidenced by the observed radioactivity in all the tissues studied within one hour of drug administration. The Cmax in the stomach (47.9 and 30.7 μg eq/g) was reached in one hour in both male and female rats, while in small intestine (166.3 and 186.1 μg eq/g) it was observed after two hours in male rats and four hours in female rats. The drug was rapidly distributed to various organs with the Cmax in the liver (186.8 and 189.8 μg eq/g) being observed by 4.7 h both in male and female rats. The Cmax in kidney (53.6 and 31.0 μg eq/g), heart (10.1 and 7.9 μg eq/g) and lungs (36.4 and 41.9 μg eq/g) was reached in seven to 10 h in both male and female rats. The Cmax in spleen (88.0 and 76.6 μg eq/g) was reached in 21 h and in brain (6.7 and 8.2 μg eq/g) it was in about 36 h. The low levels of radioactivity observed in the brain indicate that ^14^ C- pyronaridine diffuses poorly through the blood–brain barrier. The radioactivity in all the tissues gradually decreased to less than 30 μg eq/g post 48 h of drug administration. Excretion of the drug was predominantly through the urine with a peak excretion post 24 h of administration. A small amount of the drug was also excreted in the faeces and also in the breath [100].

Excretion balance investigations in the rat, following a single oral dose of ^14^ C] pyronaridine tetraphosphate at 60 mg/kg, produced an overall recovery of 93% over the 14 day collection period. A mean of 83% of the dose was excreted in faeces, with 2.6% excreted in urine, 6.3% recovered from the carcass at 14 days and the remainder in cage washings [76]. Excretion balance investigations in the dog, following a single oral dose of ^14^ C] pyronaridine tetraphosphate at 9 mg/kg, produced an overall recovery of 44% over the 14 day collection period. A mean of 36% of the dose was excreted in faeces, with 5.5% excreted in urine. The shortfall in recovery was investigated and a significant proportion of the dose was found to remain associated with the liver. The liver removed from a single animal at 6 months post-dose contained 14.3% of administered radioactivity. Other tissues contained considerably less, with the kidney being the next highest with 0.3% of administered radioactivity at 6 months post-dose [76]. Further sampling of dog liver showed a slow but steady decline in the concentration of radioactivity with 6.5% of administered radioactivity remaining in the liver at 24 months post-dose [76]. Profiling of liver extracts indicated a single major dose-related component, which was confirmed to be unchanged pyronaridine [76].

## Clinical pharmacokinetics

Some data are available regarding the clinical pharmacokinetics of pyronaridine derived from HPLC plasma assays in volunteers and patients [6,95,101]. Oral administration of 400 mg pyronaridine (6.15 mg/kg) to one healthy volunteer gave a Cmax of 76.2 ng/ml at a Tmax of one hour. The area under the curve (AUC_(0−12)_) was 662.9 ng.h/ml [101]. The drug was poorly absorbed from the tablet formulation used and drug levels were below the lower limit of quantitation by 24 h after dosing (Limit of detection = 25 ng/mL) and half-life was not estimated. [101]. A further study of pyronaridine as a single oral dose (400 mg) given to a healthy volunteer found a Cmax in plasma of 495.8 ng/ml at a Tmax of 0.5 h. The T_1/2_ was 241 h, the AUC_(0−∞)_ 51,700 ng.h.ml, clearance (CL_T_) 1.90 ml/min/kg and volume of distribution 41.2 L/kg [95]. In five Thai patients with uncomplicated malaria receiving a three-day course of oral pyronaridine tetraphosphate as a new capsule formulation 12 mg/kg the Cmax in plasma was 120 ± 30 ng/ml, Tmax was 80.0 ± 79.9 h and the AUC_0−∞_ 29,400 ± 13,100 ng.h/mL. Pyronaridine after repeat dosing was eliminated from plasma with a mean half-life of 194.8 ± 47.8 h [6]. Plasma pyronaridine profiles, in contrast to blood level profiles, do not show clear distribution and elimination profiles and thus, half-life determinations based upon plasma data may not be strictly comparable to half-lives determined from blood level data.

As pyronaridine concentrates in erythrocytes [4,32,38,93], plasma assays may underestimate pyronaridine concentrations. The pharmacokinetics of pyronaridine in blood have been investigated in patients with *P*. *falciparum* or *P. vivax* malaria using a spectrofluorometric assay. [102]. Intramuscular pyronaridine 206 mg (n = 4) was rapidly absorbed; mean (± SD) Cmax was 525 ± 104 ng/ml at Tmax 0.66 ± 0.21 h and the T_1/2α_ was 1.0 ± 0.3 h and T_1/2β_ was 63 ± 5 h. Distribution in tissues was extensive; V_c_ was 11 ± 11 L/kg, V_d(SS)_ 72 ± 33 L/kg and CL_T_ 0.9 ± 0.35 L/kg.h. The relative bioavailability of two oral formulations (600 mg) was 19 ± 7% for enteric-coated tablets (n = 3) and 32 ± 7% for capsules (n = 3) [102]. Pyronaridine pharmacokinetics for the tablets and capsules were, respectively: Cmax 130 ± 32, 255 ± 144 ng/ml; Tmax 14.0 ± 0.3, 4.72 ± 0.26 h; T_1/2β_ 65 ± 6, 63 ± 6 h; and CL_T_ 0.796 ± 0.002, 0.71 ± 0.14 kg/L.h [102]. Since blood sampling was only over 72 h, it is probable that T_1/2β_ was underestimated in this study.

The population pharmacokinetics of pyronaridine in healthy and malaria infected subjects with uncomplicated falciparum and vivax malaria after the administration of oral pyronaridine/artesunate (3:1 ratio) have been published as an abstract [103]. Pyronaridine blood concentrations were measured in nine Phase I-III clinical studies using HPLC and LCMS methods with LLOQ 5.7 ng/mL. The population pharmacokinetics are reported using data from healthy (166) and malaria infected (642) subjects. Pyronaridine pharmacokinetics data were best described by a two-compartment model with first order absorption and elimination. Malaria infection was a significant covariate for central volume of distribution and clearance. Body weight was a significant covariate for peripheral volume of distribution and clearance. After the inclusion of statistically significant covariates, the population parameter estimates of apparent clearance, central volume of distribution, peripheral volume of distribution, apparent inter- compartmental clearance and absorption rate constant (Ka) were 434 L/day, 907 L, 4,430 L, 1,120 L/day and 16.7 day^-1^, respectively. The corresponding inter-individual variability estimates for CL/F, V2/F, V3/F, Q/F and Ka were 53.6%, 103%, 29%, 28.8% and 67.5%, respectively. The elimination half-lives of pyronaridine in healthy adult subjects, adult malaria subjects were estimated to be 11.3 and 13.2 days, respectively.

### Clinical pharmacokinetics in children

Pharmacokinetic characteristics of pyronaridine, artesunate, and dihydroartemisinin were evaluated in a clinical study with tablet (6 + 2 mg/kg, 9 + 3 mg/kg, 12 + 4 mg/kg pyronaridine + artesunate respectively) and granule (9 + 3 mg/kg) formulations in Gabonese children aged two to 14 years [104]. Pyronaridine drug concentrations similarly increased in a dose dependent pattern: C_max_ and AUC rose from 85.7 ng/ml to 338.5 ng/ml and 17,623 to 35,360 ng/ml*hr, respectively. T_max_ was between 2.4 and 3.2 h and the elimination half-life ranged between 6.6 and 9.0 days. Based upon population pharmacokinetic modeling, the elimination half-life of pyronaridine in pediatric malaria subjects was estimated to be 9.6 days (103). Artesunate is rapidly converted to dihydroartemisinin *in vivo*. Mean C_max_ and AUC_INF_ levels for artesunate increased dose dependently from 92.8 ng/ml to 287.0 ng/ml and 104.3 to 232.3 ng/ml*hr, respectively. T_max_ was within a range of 0.5 to 1.0 h and t_1/2_ was between 0.5 to 1.2 h. Dihydroartemisinin showed a linear increase in C_max_ from 479.1 to 1,185.9 ng/ml and AUC values from 1,054.5 to 2,961.0 ng/ml*hr. The time to maximal drug concentrations was between 1.3 and 1.7 h after drug administration. Dihydroartemisinin showed a relatively short half-life of 0.9 to 1.2 h in the respective treatment groups. Pharmacokinetic parameters of the granule paediatric co-formulation were compared with the respective tablet formulation of the same dose strength (3 mg/kg artesunate and 9 mg/kg pyronaridine). There were no statistically significant differences in pharmacokinetic parameters except for a higher C_max_ of pyronaridine in the patient group receiving the paediatric drug formulation (168.3 ng/ml *versus* 118.5 ng/ml; p < 0.05).

## Clinical efficacy

### Monotherapy in falciparum malaria

#### Adults

Pyronaridine was approved for human use in China in 1980 at a total oral dose of 1,200 mg (or 24 mg/kg) divided into two doses on Day 0 and one dose on each of the following one or two days. Intramuscular or IV pyronaridine was given as 300 mg (or 6 mg/kg) divided into two doses eight hours apart. The main clinical studies of oral, IM and IV pyronaridine monotherapy conducted in China have been reviewed in English by Fu and Xiao and Shao and are summarized in Additional file 5[5,79,105]. The pyronaridine IM dose was later increased to 480 mg (or 9.6 mg/kg) divided into three doses once daily for three days for the treatment of chloroquine-resistant *P. falciparum*; no cases of recrudescence were seen in 10 patients after 30 days of follow up [3,71,106]. Plain tablet and enteric-coated tablet oral formulations were also tested in different dosage regimens (Additional file 5) [105]. Anecdotally, pyronaridine was used successfully to treat at least 40 cases of cerebral malaria or malaria in late pregnancy [3].

Outside China, two clinical trials of oral pyronaridine for the treatment of falciparum malaria have been reported in adults: one randomized trial in Cameroon in comparison with chloroquine and one in Thailand comparing two doses of pyronaridine (Additional file 6) [57,107]. One retrospective report using data from the Cameroon studies has also been published [108]. In addition, a case study from Indonesia reported radical cure of multidrug-resistant falciparum malaria with pyronaridine 1,200 mg with no recrudescence at Day 28 after failure of chloroquine and mefloquine [109].

The efficacy of pyronaridine monotherapy in uncomplicated falciparum malaria was compared with that of chloroquine in a randomized open-label study conducted in Cameroon (Additional file 6) [107]. Adult patients received either pyronaridine 32 mg/kg (n = 40) or chloroquine 25 mg/kg (n = 41) given in four divided doses over three days (two doses on Day 0). The clinical response at Day 14 was 100% for pyronaridine *versus* 58.5% for chloroquine (*P* = 0.0001). All patients receiving pyronaridine had a negative parasite count on or before Day 3 or positive on Day 3, but <25% of pre-treatment density plus negative there after until Day 14 compared with 18/41 (43.9%) of chloroquine-treated patients. Four cases of chloroquine failure required treatment with an alternative anti-malarial on or before Day 3. There was no significant difference in parasite clearance or fever clearance times between treatment groups for those patients that had a successful clinical and parasitological response (Additional file 6) [107]. The IC_50_ for pyronaridine against 67 *P. falciparum* clinical isolates tested was 4.82 nM, 6.7-fold more active than chloroquine against 30 chloroquine-sensitive isolates (IC_50_ 32.5 nM) and 73-fold more active than chloroquine against 39 chloroquine-resistant isolates (IC_50_ 354 nM) [107].

Looareesuwan *et al.* evaluated the efficacy of two dosing regimens of pyronaridine in a non-randomized, open-label study of 101 adult Thai patients with uncomplicated falciparum malaria (Additional file 6) [57]. Patients received either 1,200 mg in four divided doses over three days, two doses on Day 0 (n = 69) or 1,800 mg in six divided doses over five days, two doses on Day 0 (n = 32). Based on the mean weight of subjects enrolled, the 1,200 mg dose given in the Thailand study is equivalent to a mean dose of 25.7 mg/kg and the 1,800 mg dose to a mean dose of 36.4 mg/kg. Clinical success at Day 28 was achieved in 38/60 (63.3%) evaluable patients for the 1,200 mg dose and 23/26 (88.4%) for the 1,800 mg dose (*P* < 0.025). Recrudescence occurred between Days 13 and 28 (median 24 days) in the 1,200 mg group and between Days 11 and 28 (median 17 days) in the 1,800 mg group. Sixteen patients in the 1,200 mg group and 11 in the 1,800 mg group had at least one parasite count greater than their initial count [57]. There were no significant differences in time to clear fever or parasites between the two treatment groups (Additional file 6) [57]. The time to clear 50% and 90% of parasites were 68 h and 152 h for the 1,200 mg group and 70 h and 120 h for the 1,800 mg group, respectively. The pyronaridine mean pre-treatment IC_50_ for 10 patients that were cured was 15.69 ± 3.82 nM compared with 22.98 ± 12.1 nM in 10 patients who had recrudescence. The authors suggested that there was a relationship between treatment result and parasite sensitivity to pyronaridine. However, there was no evidence of the development of resistance after unsuccessful treatment with pyronaridine: the initial pyronaridine mean IC_50_ was 23.39 ± 12.71 nM *versus* 22.96 ± 11.54 nM after recrudescence in *P. falciparum* isolates from nine patients for whom paired data were available [57].

In contrast to the Cameroon study, recrudescences were seen in Thailand; fever clearance and parasite clearance times were also extended by 47.9−50.5 h and 7.6−9.9 h, respectively [57,107]. These differences in efficacy between Cameroon and Thailand were not unexpected. In this region of Thailand the high frequency and degree of resistance to chloroquine, sulphadoxine and pyrimethamine and the high prevalence of multi-resistant *P. falciparum*, even in the mid-1990s, presented a difficult challenge for new therapy [110]. The 88% cure rate for the 1,800 mg dose of pyronaridine compares favourably with contemporary anti-malarial monotherapy studies also conducted at the Bangkok Hospital for Tropical Diseases: 88% for artesunate (600 mg/5 days), 74% for oral artemether (500 mg/5 days) and 81% for mefloquine (1250 mg) [57]. In Cameroon, Day 28 recrudescence rates around the time of the study were reported as 22/131 (16.8%) for chloroquine, 4/66 (6.1%) for sulphadoxine−pyrimethamine and 0/59 for amodiaquine [108]. When comparing IC_50_s obtained in the two clinical studies, pyronaridine was 3.3-fold more active against Cameroon isolates than against those from Thai patients that were cured and 4.8-fold more active against those from Thai patients who had recrudescence [57,107].

#### Children

One study of oral pyronaridine for the treatment of uncomplicated falciparum malaria in children has been conducted in Cameroon (Additional file 6) [111]. Patients were aged between five and 15 years old with a mean parasitaemia of 103,000 asexual parasites/μL of blood (range 7590−609,600 parasites/μL). All 41 patients receiving pyronaridine (32 mg/kg, four divided doses, two on Day 0) were clinically and parasitologically cured at Day 7 and Day 14. Chloroquine 25 mg/kg (10 mg/kg Day 0 and 1, 5 mg/kg Day 2) was significantly less effective than pyronaridine for both clinical response (*P* = 0.001) and parasitological response at Day 14 (*P* = 0.0001). In the chloroquine group, 5/40 (12.5%) patients required alternative anti-malarial therapy on or before Day 3. There was no significant difference in fever or parasite clearance times between the two therapies in those patients that had a successful clinical and parasitological response (Additional file 6) [111]. The geometric mean IC_50_ of pyronaridine was 6.89 nM (n = 25, range 1.95−34.2 nM) for patients treated with pyronaridine and 5.69 nM (n = 27, range 0.8−16.6 nM) for those who received chloroquine. For patients in the pyronaridine group, 23/40 (57.5%) of the isolates tested were chloroquine-resistant (chloroquine IC_50_ >100 nM). Clinical and parasitological efficacy of pyronaridine was demonstrable against all of these chloroquine-resistant isolates.

## Combination therapy in falciparum malaria

Pyronaridine plus sulphadoxine−pyrimethamine or sulphadoxine−primaquine

Based on *in vitro* data and clinical studies, the use of pyronaridine in combination therapy was recommended in China for use in areas of endemic chloroquine-resistant *P. falciparum*[56,66,71,112-116]. Additional file 7 summarizes the clinical studies conducted in China for pyronaridine−sulphadoxine−pyrimethamine and pyronaridine−sulphadoxine−primaquine in Hainan and Yunnan Provinces, respectively [66,71].

A small study (n = 101) was conducted in Hainan province, China, where chloroquine resistant *P. falciparum* is endemic, where patients with acute symptomatic falciparum malaria were treated sequentially with pyronaridine−sulphadoxine−pyrimethamine orally, with one of three dosage regimens (Additional file 7) [66]. The combination cleared fever in 30.1−38.4 h and parasitaemia within 41.9−48.7 h; there were no recrudescences within four weeks after therapy (Additional file 7) [66].

## Pyronaridine plus nitroquine

Pyronaridine plus nitroquine was also studied in Hainan; comparators included pyronaridine or piperaquine monotherapy and piperaquine plus nitroquine [117]. There was no improvement with pyronaridine/nitroquine combination therapy in recrudescence rates, or times to fever and parasite clearance *versus* pyronaridine monotherapy (Additional file 7) [117].

## Pyronaridine plus artemisinin derivative combination therapy

### Pyronaridine plus dihydroartemisinin

A double-blind study in China investigated the efficacy of pyronaridine (1600 mg, n = 25) or dihydroartemisinin (DHA, 640 mg, n = 24) alone or in combination (800 + 300 mg, n = 32) in uncomplicated falciparum malaria (Additional file 7) [118]. The recrudescence rate at Day 28 was 0% for pyronaridine 4.2% for DHA and 0% for the combination. Fever resolution was significantly more rapid with the combination (35.7 ± 24.7 h) than with DHA alone (52.6 ± 38.9 h, *P* < 0.01). Time to parasite clearance was significantly faster with the combination (23.8 ± 10.1 h) than with pyronaridine alone (49.4 ± 20.3 h, *P* < 0.01). Gametocyte carriage was 20.0% for the combination, 16.7% for DHA alone and 60.9% for pyronaridine alone, which was significantly higher than for the combination (*P* < 0.01) [118]. These early results showed promise for pyronaridine combination therapy with artemisinins and indicated that a lower dose of both compounds could be used effectively *versus* the monotherapy doses.

### Pyronaridine plus artesunate combination: tablet formulation

Pyronaridine-artesunate has been developed as a fixed-dose combination therapy by Shin Poong Pharmaceutical Ltd, South Korea, with the tetraphosphate pyronaridine salt in a 3:1 ratio with artesunate as an oral once daily treatment for three days, for uncomplicated *P. falciparum* malaria and for the blood stages of *P. vivax* malaria in adult and paediatric patients. A conference report is available for a double-blind, multicentre, randomized Phase II study including 477 adults in Africa and SE Asia [119].

One Phase II study, conducted in Gabon, has been reported [104]. Two fixed-dose pyronaridine-artesunate formulations (tablets and granules) were compared in children (two to 14 years old) for the treatment of uncomplicated falciparum malaria. An open-label dose escalation study design was used, recruiting 15 patients sequentially in each of four treatment cohorts. The combination was administered once daily for three days as co-formulated tablets at the following dose levels: 2:6 mg/kg, 3:9 mg/kg, and 4:12 mg/kg artesunate and pyronaridine, respectively. Additionally, a paediatric granule co-formulation was investigated at the medium dose strength (3:9 mg/kg) in a fourth cohort. The combination showed a good tolerability profile at all dose levels and there were no safety concerns. Pharmacokinetic analysis revealed a dose dependent increase in C_max_ and AUC values and a comparable relative bioavailability of the granule co-formulation. At all dose levels efficacy of pyronaridine artesunate combination therapy was 100% in the per protocol analysis on Day 28 after PCR correction [104].

A Phase III programme with fixed-dose pyronaridine-artesunate included four phase III pivotal studies; two studies in adults and children in *P. falciparum* malaria *versus* artemether-lumefantrine in one study and mefloquine + artesunate in the other study, one study in adults and children in *P. vivax* malaria *versus* chloroquine [120] and one study in children only in *P. falciparum* malaria with the granule formulation *versus* artemether-lumefantrine. Overall, in the clinical programme, a total of 2,815 patients were treated with pyronaridine-artesunate, including 1,528 adults (≥ 18 years), 401 patients 12–18 years and 886 children (< 12 years) [120]. Pyronaridine-artesunate showed high levels of efficacy in adults and children malaria patients. In both populations pyronaridine-artesunate and the comparator drugs were well tolerated. The rate and type of adverse event were generally comparable between the various treatment groups and were of mild or moderate severity [104,120].

A pivotal, multicentre, randomized, comparative, parallel group, double-blind, double-dummy study compared the efficacy and safety of once a day pyronaridine-artesunate with twice a day lumefantrine-artemether in uncomplicated *P. falciparum* malaria in children and adults [121]. A total of 1,272 patients were randomized to treatment, 84.9% from Africa and 15.1% from Asia. For the primary endpoint, Day-28 PCR-corrected adequate clinical and parasitological response (ACPR) in the per protocol population, pyronaridine-artesunate was non-inferior to lumefantrine-artemether. Both treatments were highly efficacious with cure rates >99% and no early clinical failures. These results are consistent with the high activity observed for pyronaridine-artesunate against African *P. falciparum* isolates and 100% cure rates from the previous pyronaridine-artesunate clinical study [104]. Day-28 crude ACPR (per protocol population) and Day-42 PCR-corrected and crude ACPR (per protocol and intent to treat population) were superior with pyronaridine-artesunate *versus* lumefantrine-artemether. Kaplan–Meier analysis confirmed an important difference in re-infection rate between treatment groups through Day 28 and 42. Parasite clearance was more rapid with pyronaridine-artesunate *versus* lumefantrine-artemether (*p* < 0·001), with the greatest difference seen before Day 2. There was no difference in fever clearance time between the treatment groups.

A further pivotal, multicentre, randomized, comparative, parallel group, open-label study compared pyronaridine-artesunate and a loose combination of mefloquine + artesunate in uncomplicated *P. falciparum* malaria in children and adults. Overall, 1,271 patients were included in the study, 81% from Asia, 19% from Africa. Day-28 PCR-corrected ACPR in the per protocol population were 99.2% for pyronaridine-artesunate and 98.1% for mefloquine + artesunate. Pyronaridine-artesunate was non-inferior to mefloquine + artesunate; treatment difference 1.1% (95% CI −0.2, 3.1; p = 0.106). ACPR were ≥ 95.7% across individual study centers for both study treatments. In 211 patients in the region of Cambodia where extended parasite clearance times are reported, parasite clearance time for both treatments was prolonged compared with other countries (P < 0.001, Kaplan–Meier); median time to parasite clearance was twice as long (64 *versus* 31–32 hours) [122].

### Pyronaridine plus artesunate combination: paediatric formulation

A phase III comparative, double-blind, double dummy, randomized, non-inferiority, multicentre clinical study was conducted in paediatric patients to assess the efficacy and safety of a granule fixed dose formulation of oral pyronaridine-artesunate (60:20 mg) *versus* artemether-lumefantrine tablet (20:120 mg) in 535 patients (mean 5.0 years (0 to 12 years); mean 16.6 kg (6–24.9 kg)) with acute uncomplicated *P. falciparum* malaria. Patients from seven sites in Africa and South East Asia were randomized to receive a three-day course of either pyronaridine-artesunate once a day or artemether-lumefantrine twice a day [121]. In the per protocol population, the PCR-corrected ACPR was 97.6% and 98.8% at Day 28 and 79.5% and 82.6% at Day 42 with pyronaridine-artesunate and artemether-lumefantrine respectively. Results demonstrate non-inferiority of pyronaridine-artesunate to artemether-lumefantrine at Day 28 with a treatment difference −1.2% (95% CI −3.6, 2.1; p = 0.373). The crude ACPR was 90.2 and 89.2 at Day 28 (non-inferiority) and 77.4% and 80.2% at Day 42 with pyronaridine-artesunate and artemether-lumefantrine (non-inferiority), respectively. Median time until parasite clearance was 24.1 h and 24.2 h for pyronaridine-artesunate and artemether-lumefantrine respectively [121].

## Non-falciparum malaria

### *Plasmodium ovale* and *Plasmodium malariae* malaria

Pyronaridine efficacy was investigated in 22 patients with *P. ovale* (n = 10) and/or *P. malariae* infection (n =16) in Cameroon [74]. Mean fever and parasite clearance times were 49.8 h and 33.5 h, respectively. All patients were apyretic by Day 4 and all had clinical and parasitological cure at Day 14 [74]. No gametocytes were found on Day 3 [74].

### *Plasmodium vivax* malaria

Pyronaridine has been evaluated both alone and in combination with primaquine for efficacy against *P. vivax* (Additional file 8) [5,79,123,124]. The efficacy of pyronaridine 1200 mg over three days oral monotherapy against *P. vivax* was similar to that of chloroquine [79]. Intramuscular pyronaridine results in marginally faster parasite clearance than with oral and IV administration.

Pyronaridine has also been evaluated in combination with short-course (three- or four-day) primaquine [123,124]. It had acceptable efficacy, though comparator regimens including primaquine for eight days were the most effective among those tested at preventing relapse (Additional file 8) [123,124].

The fixed dose pyronaridine-artesunate combination tablet has been tested as an oral once daily treatment over three days for blood stage of *P. vivax* malaria [125]. A pivotal phase III multicentre, randomized, double-blind, double-dummy, parallel-group comparative, non-inferiority trial was conducted for curative treatment of *P. vivax* malaria. The primary objective of this clinical study was to compare the efficacy and safety of the fixed combination of pyronaridine-artesunate with that of standard chloroquine therapy in adults and children with acute, uncomplicated *P. vivax* malaria. This trial included five centres across Cambodia, Thailand, India, and Indonesia. In a double-dummy design, patients (aged >3–≤60 years) with microscopically confirmed *P. vivax* mono-infection were randomized (1:1) to receive pyronaridine-artesunate (target dose 7.2:2.4 mg/kg to 13.8:4.6 mg/kg) or chloroquine (standard dose) once daily for three days. For patients who completed the study up to Day 28 and who had normal glucose-6-phosphate dehydrogenase activity, a 14-day course of primaquine (15 mg/day) was administered starting on Day 28, to complete their radical cure. Each treatment group included 228 randomized patients. Outcomes for the primary endpoint, Day-14 cure rate in the per-protocol population, were 99.5%, with pyronaridine-artesunate and 100% with chloroquine. Pyronaridine was non-inferior to chloroquine: treatment difference −0.5% (95% CI −2.6, 1.4), i.e. the lower limit of the two-sided 95% CI for the treatment difference was greater than −10%. Pyronaridine-artesunate cure rates were non-inferior to chloroquine for Days 21, 28, 35 and 42. Parasite clearance time was shorter with pyronaridine-artesunate (median 23.0 h) *versus* chloroquine (32.0 h; p < 0.0001), as was fever clearance time (median 15.9 h and 23.8 h, respectively; p = 0.0017). Kaplan-Meier estimates of post-baseline *P. falciparum* infection incidence until Day 42 were 2.5% with pyronaridine-artesunate, 6.1% with chloroquine (p = 0.048, log-rank test). Post-baseline *P. vivax* or *P. falciparum* infection incidence until Day 42 was 6.8% and 12.4%, respectively (p = 0.022, log rank test) [125].

## Pyronaridine plus artesunate combination: pooled analyses

Integrated analysis of efficacy were performed on pooled data from the four Phase III pivotal studies; the Phase III clinical efficacy database included 1,701 patients from Asia and 1,833 patients from Africa [120].

In each of the three Phase III *P. falciparum* studies, non-inferiority of pyronaridine-artesunate *versus* the comparator (i.e., artemether-lumefantrine or mefloquine + artesunate) was demonstrated as primary endpoint for PCR-corrected ACPR on Day 28 (per protocol and ITT populations). The percentage of subjects with gametocytes gradually decreased to zero or near zero over time in the pyronaridine-artesunate, mefloquine + artesunate and artemether-lumefantrine treatment groups.

In the study in subjects with *P. vivax* malaria, non-inferiority of pyronaridine-artesunate compared with chloroquine was demonstrated with respect to the crude cure rate on Day 14 (per protocol population). Non-inferiority was also demonstrated for both PCR-corrected and crude ACPR at all time points (for both PP and ITT populations).

Pyronaridine-artesunate showed high levels of efficacy in *P. falciparum* and *P. vivax* adults and children malaria patients and similar efficacy was observed in Asia and Africa. Pyronaridine-artesunate showed high cure rates, similar to those of the current standard of care therapies, along with rapid clearance of parasitaemia and most malaria-related symptoms, coupled with prevention of recrudescence. The relatively long half-life of pyronaridine could explain the observed prophylactic effect up to D42 [120].

## Tolerability

### Pyronaridine monotherapy

Studies in China found pyronaridine to be generally well tolerated; around 38% of patients experienced adverse events *versus* 56% for chloroquine [79]. Adverse events following oral administration were mild and usually resolved within two days of starting therapy. The most common adverse events after oral pyronaridine therapy in many cases are similar to the symptoms of malaria, ie dizziness, nausea, vomiting and abdominal discomfort [79]. There were also some reports of palpitations and allergic skin reaction [3]. Transient ECG changes have also been noted at higher pyronaridine doses [3,5]. There is some evidence that tolerability was improved with a plain tablet formulation at an 8 mg/kg total dose (5 mg/kg Day 0, 3 mg/kg Day 2) *versus* an enteric-coated tablet at 12 mg/kg (4 mg/kg bid Day 0, 4 mg/kg Day 2); 18.8% (6/32) and 28.1% (9/32) patients experienced an adverse event, respectively.

Gastrointestinal effects were mostly absent after IM administration, though there was some local injection-site irritation without necrosis. Intravenous administration was mostly without adverse effects, though nausea, palpitation, diarrhoea and abdominal pain were noted in a few patients [79]. At least 10 pregnant malaria patients were treated successfully with pyronaridine during their mid- to late-trimester with no known adverse effects [5].

There were some differences in the adverse event profile for pyronaridine monotherapy between trials conducted in Cameroon and Thailand (Additional file 9) [57,107]. The most common treatment emergent adverse events reported in African adults were abdominal pain (32.5%), diarrhoea (25.0%) and pruritus (17.5%) and in Thailand were headache (36−38%), dizziness (28−33%) and nausea (11−13%). Pruritis is a known adverse reaction seen in African patients to many anti-malarials, particularly chloroquine. The incidence of pruritus with chloroquine was approximately 2.5x that of pyronaridine in the Cameroon study [107]. A small study in Cameroon in patients with *P. malariae* or *P. ovale* infection also reported pruritis with pyronaridine in 3/20 (15.0%) patients, and 9/20 (45.0%) had mild gastrointestinal symptoms [74].

In the Cameroon study, haemoglobin was below 8 g/dL on Day 7 in one patient treated with pyronaridine and three receiving chloroquine [107]. Serum transaminases were increased *versus* baseline in seven patients treated with pyronaridine and four treated with chloroquine, values were normal by Day 14 in all cases. Total bilirubin was slightly elevated in five patients receiving pyronaridine on Day 7 [107].

In the Thailand study, one patient receiving 1,200 mg pyronaridine and six receiving 1,800 mg pyronaridine had increased transaminases at Day 7; all cases resolved within four to five weeks after therapy start [57]. There were no other laboratory changes of note.

In a study conducted in Cameroon, the most common treatment emergent adverse events with pyronaridine in children (n = 41) were abdominal pain (22.0%), diarrhoea (12.2%) and headache (9.8%) (Additional file 9) [111]. Pruritis occurred in 4.9% of patients receiving pyronaridine *versus* 47.5% of those receiving chloroquine (n = 40). Between Days 0 and 7 there were significant (*P* < 0.05) decreases in mean neutrophils, total bilirubin, conjugated bilirubin and blood urea and significant increases in mean lymphocytes, eosinophils, reticulocytes and platelets in both the pyronaridine and chloroquine-treated groups [111]. These changes were similar between the two treatment groups. There was no significant variation in mean values for haemoglobin, white blood cell count (WBC), serum transaminases, alkaline phosphatase or serum creatinine in either treatment group [111]. There was an inversion of the neutrophil:lymphocyte differential count with both pyronaridine and chloroquine by Day 7, though little change inWBC. In some patients, the absolute eosinophil count increased approximately four-fold with both pyronaridine and chloroquine. There was no evidence that pyronaridine inhibits erythropoiesis or WBC maturation. Three children receiving pyronaridine had two to three-fold increases in serum aspartate aminotransferases on Day 7 *versus* baseline [111].

## Artemisinin-based combination therapy

### Pyronaridine plus dihydroartemisinin

In a small study of pyronaridine (32 mg/kg, n = 25) or DHA (6 mg/kg, n = 20) alone or in combination (16/6 mg/kg, n = 32) there were no serious adverse events in any group [118]. Mild headache and nausea were observed in all three treatment groups. No changes in blood chemistry and urinalysis were observed. All adverse events disappeared one to two days after the end of treatment [118].

### Pyronaridine plus artesunate combination: tablet formulation

A Phase II study in adults with *P. falciparum* malaria investigated the safety of fixed dose pyronaridine artesunate at three doses: 6 + 2 (n = 160), 9 + 3 (n = 157) or 12 + 4 mg/kg (n = 159) and limited data have been reported in abstract form [119].

In the Phase III pivotal, multicentre, randomized, comparative, parallel group, double-blind, double-dummy study in a total of 1,272 patients the efficacy and safety of once a day pyronaridine-artesunate with twice a day lumefantrine-artemether in uncomplicated *P. falciparum* malaria in children and adults were compared [121]. Safety findings with pyronaridine-artesunate were generally consistent with the safety profile for pyronaridine and artesunate given as monotherapy in Africa and Thailand Similar proportions of patients experienced at least one adverse event in both treatment groups; 60% for pyronaridine-artesunate and 57% for lumefantrine-artemether. Adverse events thought by the investigator to be treatment-related occurred in a similar proportion of pyronaridine-artesunate-treated (32.4%) and lumefantrine-artemether-treated (29.1%) patients. There were no deaths during the study. Serious adverse events were reported for 3 [0.4%] subjects receiving pyronaridine-artesunate, and 2 [0.5%] subjects receiving lumefantrine artemether, none of which was considered by the investigator to be related to study drug. The only safety finding requiring further investigation was transient increases in hepatic enzymes with pyronaridine-artesunate; more patients experienced >3ULN rises in ALT ± AST. However, these were accompanied in two cases only by a rise of bilirubin >2ULN and without a concomitant significant rise in alkaline phosphatase. Values for AST and ALT were close to or within normal limits by Day 28.

In the pivotal, multi-centre, randomized, comparative, parallel group, open-label study comparing pyronaridine-artesunate and mefloquine + artesunate, 1,271 patients were included. Similar proportions of patients experienced at least one adverse event in both treatment groups; 45.9% for pyronaridine-artesunate and 44.9% for mefloquine + artesunate [122]. Adverse events occurring in > 5% of patients were headache and myalgia with pyronaridine-artesunate and headache and dizziness with mefloquine + artesunate. Adverse events thought by the investigator to be treatment-related occurred in a similar proportion of pyronaridine-artesunate-treated (18.0%) and mefloquine + artesunate-treated (22.2%) patients. Adverse events occurring in > 2% of patients were headache and increased alanine aminotransferase with pyronaridine-artesunate, and headache, dizziness and anaemia with mefloquine + artesunate. There were no deaths during the study. Serious adverse events were reported for six (0.7%) patients receiving pyronaridine-artesunate and three (0.7%) receiving mefloquine + artesunate. Only two serious adverse events, both in the mefloquine + artesunate group, were considered treatment-related by the investigator; convulsion and grand mal convulsion. There were no clinically concerning changes in haematology parameters during the study and no differences between treatment groups. Mean alanine aminotransferase was increased in the pyronaridine-artesunate group at Day 3 (+6.4 U/L) and Day 7 (+12.8 U/L). Three pyronaridine-artesunate-treated patients had peak alanine aminotransferase ≥ 3 times the upper limit of normal (ULN) plus peak bilirubin ≥2 ULN; one patient also had aspartate aminotransferase ≥3 ULN at Day 42. Alanine aminotransferase and aspartate aminotransferase values were within normal limits by Day 28 for one subject; peak values were at the final recorded evaluation for the other two subjects. Other clinical chemistry values were similar between treatment groups.

The fixed dose pyronaridine-artesunate combination tablet has been tested as an oral once daily treatment for three days for blood stage of *P. vivax* malaria in a pivotal Phase III multi-centre, randomized, double-blind, double-dummy, parallel-group comparative, non-inferiority trial [125]. In the pyronaridine-artesunate group, 92/228 (40.4%) patients experienced a treatment-emergent adverse event of any cause compared with 72/228 (31.6%) in the chloroquine group. Increased transaminases were more common in the pyronaridine-artesunate group (2.2%) *versus* the chloroquine group (0%). All adverse events in the pyronaridine-artesunate group and the majority (70/72; 97.2%) in the chloroquine group were of mild-to-moderate severity. Adverse events deemed study drug related by the investigator occurred in 27/228 (11.8%) patients in the pyronaridine-artesunate group and 23 (10.1%) in the chloroquine group. There were no deaths during the study. Two patients, both in the pyronaridine-artesunate group, had serious adverse events (one pyrexia, one typhoid fever); neither was considered drug related by the investigator. Two patients, both in the chloroquine group, had adverse events leading to drug discontinuation and study withdrawal (one vomiting plus fatigue, one vomiting); all considered possibly related to drug treatment by the investigator. No clinically meaningful difference in change from baseline in haemoglobin was observed between patients with or without phenotypic G6PD deficiency. Other haematology laboratory findings (increases in platelets, eosinophils, and lymphocytes and a decrease in neutrophils) were of similar magnitude in both treatment groups. Biochemistry laboratory observations were generally similar in both treatment groups, with the exception of alanine transaminase (ALT) and aspartate transaminase (AST). From Day 3 to the end of the study, 3/228 (1.3%) patients in the pyronaridine-artesunate group had peak ALT > 5x the upper limit of normal (ULN); two (0.9%) with peak ALT > 10 × ULN. Total bilirubin values were within normal limits for both of these subjects throughout the study. No patients in the chloroquine group had ALT > 5xULN. Post-treatment AST was > 5 × ULN in one patient in the chloroquine group and > 10 × ULN in one patient from the pyronaridine-artesunate group; total bilirubin was within normal limits. Values for AST and ALT were close to or within normal limits by Day 28. No patient had peak ALT or AST > 3 × ULN plus total bilirubin > 2 × ULN during the study.

### Pyronaridine plus artesunate combination: paediatric formulation

One study, conducted in Gabon, included children aged two to 14 years old with the tablet formulation of fixed-dose pyronaridine-artesunate at three different ratios 6:2, 9:3 and 12:4 mg/kg or a paediatric granule formulation of fixed-dose pyronaridine-artesunate at 9:3 mg/kg; both formulations were given once daily for three days [104]. In this study, between 70% and 90% of patients experienced at least one adverse event, one third of which (20–36%) was judged as being at least possibly study drug related (Additional file 10). The majority of study drug-related adverse events was observed in the category of gastrointestinal disorders, including patients experiencing vomiting (n = 4), abdominal pain (n = 4), diarrhoea (n = 1), and nausea (n = 1). Other possibly drug-related adverse events were rare. No study drug-associated abnormal electrocardiographic finding occurred in this clinical trial. All adverse events were of mild or moderate intensity and there was no severe or life threatening adverse events or increase in the rate of events based on dose. Two serious adverse events occurred in the lowest dose group (6 mg/kg pyronaridine + 2 mg/kg artesunate) and were not study drug related. In this study there was a modest decrease in median haemoglobin levels in the first 72 h of treatment with complete recovery by Day 7. A general rise in platelet count and eosinophils was noted. There were no clinically significant changes in the biochemistry parameters during the course of this study.

### Pyronaridine plus artesunate combination: pooled analyses

In an integrated safety analysis of the pyronaridine-artesunate Phase III studies, 57.2% of pyronaridine-artesunate patients reported at least one adverse event after baseline [120]. Pyronaridine-artesunate and the comparator drugs were well tolerated. Overall, the rate and type of adverse event were generally comparable between the various treatment groups and were of mild or moderate severity. There were no deaths and few SAEs (0.6% in pyronaridine-artesunate patients) and most of them were not related to pyronaridine-artesunate. Overall, no changes in vital signs were observed except for a fall in heart rate immediately after treatment, probably reflecting the clearance of fever. The pattern of changes seen in clinical laboratory parameters was consistent with acute malaria and its resolution after treatment. All biochemistry changes were transient and reversible and not associated with any symptomatology. Pyronaridine-artesunate was not associated with any increased risk of haematologic adverse events.

The hepatic data were reviewed by an independent data monitoring committee (IDMC) that consisted of six members, including three international experts in Drug Induced Liver Injury (DILI). The committee concluded that a three-day treatment with pyronaridine-artesunate can cause transient rises in ALT concentration in a small subset of patients. However, the early onset (Day 3–7), dose response trend, and rapid resolution were all consistent with direct low-level toxicity. The IDMC commented that with hepatotoxic drugs, serious idiosyncratic hepatotoxicity typically begins after weeks or months of treatment, like described with amodiaquine, another Mannich base [126]. These findings, combined with the fact that pyronaridine-artesunate is given for only three days, suggest that the risk of progressive liver injury to subjects receiving pyronaridine-artesunate is very lowA detailed review of ECGs from patients who received pyronaridine-artesunate was conducted by a single, independent observer [127]. ECGs (n = 2,371) were reviewed from eight sites in Asia and Africa in 477 adult patients (15–60 years, mean age: 28 years) and 228 ECGs from one site in Africa in 60 paediatric patients (two-14 years, mean age: six years). ECGs were assessed for waveforms (P, QRS and T as well as the presence of U waves), rhythm (abnormal rhythms as well as tachycardia and bradycardia) the evidence of abnormalities of PR, QT, and ST segments and ECG clinically significant abnormality. In the adult study, only 2% of the total number of ECGs were considered clinically significant abnormal, with no evidence of any increased risk of QTc elongation and only 0.13% having a QTc >450 msec (all of which were < 465 msec). There were no differences with dose of pyronaridine-artesunate and generally no changes from baseline with the exception of bradycardia (HR < 55 bpm), also being the most commonly reported finding; Day 1 (5.2%) and Day 2 (7.5%) with similar frequency between Africa and Asia, being in line with literature reports [128]. In those under 14 years treated, the most frequent abnormality reported was sinus arrhythmia, which is commonly reported in children of this age. In summary the use of pyronaridine-artesunate was not associated with either significant ECG changes or increased risk of QTc prolongation.

## Current status

Pyronaridine tetraphosphate-artesunate (PYRAMAX®) is being co-developed by the Medicines for Malaria Venture (MMV) and Shin Poong Pharm Co Ltd (Republic of Korea) for the treatment of acute uncomplicated *P. falciparum* and blood stage *P. vivax* malaria with a fixed-dose combination tablet and granule formulation for paediatric administration. MMV is a not-for-profit organization, operating as a public−private partnership seeking to discover, develop and deliver new anti-malarial drugs. A complete suite of toxicology and safety pharmacology studies and a full clinical programme has been undertaken. Pivotal Phase III comparative clinical trials were completed in 2009, to include over 3,000 children, adolescents and adults across sub-Saharan Africa and South East Asia in more than 18 countries. Positive Opinion was received by the European Medicines Agency under the Article 58 procedure in March 2012. Shin Poong received approval from the Korean Federal Drug Agency in August 2011 and this will be followed by national regulatory submissions and WHO procedures.

## Conclusion

Pyronaridine has shown value as a therapeutic partner to use in artemisinin combination therapy. Pyronaridine brings high efficacy, including against chloroquine and amodiaquine-resistant strains, and the reassurance of many years of successful use in China as monotherapy and in combination with other anti-malarials, without the development of widespread drug resistance. To date no serious toxicities have been reported for pyronaridine.. Notably, pyronaridine in combination with artemisinins appears to reduce the development of resistance *in vitro*. Also, studies *in vivo* animal models indicate a synergistic effect between pyronaridine and artemisinins against parasites resistant to one or both components, restoring efficacy against these strains. Consequently, pyronaridine represents an ideal candidate for combination therapy with artemisinin derivatives, such as artesunate. Pyronaridine also appears to be well tolerated in clinical studies [57,74,104,107,111,120]. Clinical studies of the combination of pyronaridine tetraphosphate and artesunate are encouraging and show it to be a promising new artemisinin combination therapy for the treatment of both *P. falciparum* and P. *vivax* malaria in adult, children and infant populations [104,121].

## Abbreviations

ACT, Artemisinin-based combination therapy; Bid, Twice daily; ECG, Electrocardiogram; ED50, The amount of material required to produce a specified effect in 50% of a population; IC50 IC90, IC99 dose at which 50, 90 or 99% of parasites are inhibited, Respectively; IG, Intragastric; IM, Intramuscular; IP, Intraperitoneal; IV, Intravenous; LD50, Lethal dose of 50% of test population; MLD, Minimal lethal dose; QD, Once daily; SC, Subcutaneous; ALT, Alanine aminotransferase; AST, Aspartate aminotransferase.; Eq/g Pyronaridine tetraphosphate, 910.03 g.mol-1 ; Pyronaridine, 518.05 g.mol-1 ; Log D, 1.7 at pH 7.4.

## Competing interests

Dr CS Shin is former Head of Pyramax Development at Shin Poong Pharmaceuticals (retired). Other authors declare that they have no competing interests.

## Authors’ contributions

SLC, SD, SAB, LF and CSS drafted the manuscript. All authors read and approved the final manuscript.

## Supplementary Material

Additional file 1 **Comparative activity of pyronaridine against chloroquine-susceptible and -resistan**t ***P. falciparum*field isolates from SE Asia and Africa.** Click here for file

Additional file 2 **Comparative IC**_**5**0_** for drug-resistant and -sensitive strains of* P. falciparum.*** Click here for file

Additional file 3 Acute toxicity studies with pyronaridine: summary of main findings. Click here for file

Additional file 4 Sub-acute toxicity studies with pyronaridine: summary of main findings. Click here for file

Additional file 5 **Efficacy of pyronaridine monotherapy in patients with *P. falciparum *malaria: studies conducted in China.** Click here for file

Additional file 6 Oral monotherapy with pyronaridine in the treatment of falciparum malaria: International studies. Click here for file

Additional file 7 **Efficacy of oral pyronaridine combination therapy in patients with *P. falciparum *malaria.** Click here for file

Additional file 8 **Clinical studies conducted in China of pyronaridine alone and in combination with primaquine in patients with *P. vivax *malaria.** Click here for file

Additional file 9 Treatment-emergent adverse events with pyronaridine oral monotherapy in the treatment of falciparum malaria. Click here for file

Additional file 10 Treatment-emergent adverse events with fixed-dose pyronaridine-artesunate in the treatment of falciparum malaria in children. Click here for file
