# Supplementary material for: Review of pyronaridine anti-malarial properties and product characteristics
Source: Malar J. 2012 Aug 9;11:270. doi: 10.1186/1475-2875-11-270 (PMC3483207; doi:10.1186/1475-2875-11-270)
Supplement: Additional file 1 — Comparative activity of pyronaridine against chloroquine-susceptible and -resistant P. falciparumfield isolates from SE Asia and Africa. [file 1475-2875-11-270-S1.doc]

**Additional file 1.** Comparative activity of pyronaridine against chloroquine-susceptible and -resistant *P. falciparum* field isolates from SE Asia and Africa .

| **Origin[reference]** | **N** | **IC_50_, nM (95% CI)** | | | | | | | | | |
| --- | --- | --- | --- | --- | --- | --- | --- | --- | --- | --- | --- |
|  |  | **PRN** | **CLQ** | **Q** | **MQ** | **AMQ** | **APQ** | **HF** | **PYR** | **ATM** |  |
| Eastern Thailand | 30^a^ | 8.4 (6.64−10.7) | 316  (250−400) | 388  (329−455) | 6.97  (5.52−8.87) | 12.7  (10.6−15.1) | − | − | 11800  (9160−15200) | − |  |
| Northern Thailand | 7^b^ | 10.1  (6.78−15.0) | 167  (111−250) | 248  (188−328) | 5.48  (3.42−8.76) | 7.29  (4.5−11.8) | − | − | 1980  (1550−2520) | − |  |
| Africa, CLQ-S | 12 | 7.7  (6.2−9.6) | 31.3  (25.8−38.0) | − | − | 20.8^c^  (17.5−24.9) | 13.1  (11.4−15.0) | − | − | − |  |
| Africa, CLQ-R | 19 | 8.4  (6.8−10.3) | 366  (293−459) | − | − | 59.7^c^  (50.7–70.1) | 15.4  (13.1−18.3) | − | − | − |  |
| Cameroon, CLQ-S | 45 | 5.15  (4.18−6.35) | 35.6  (30.6−41.5) | 108  (87.1−134) | 7.38  (5.79−9.40) | − | − | 1.32  (1.04−1.66) | − | 2.37  (1.91−2.94) |  |
| Cameroon, CLQ-R | 74 | 4.92 (4.14−5.86) | 337  (305−372) | 239  (199−287) | 8.47  (6.78−10.6) | − | − | 1.22  (1.04−1.44) | − | 1.61  (1.30−2.00) |  |
| Senegal, CLQ-S | 80 | 2.9  (2.3−3.5) | 35.7  (30.2−40.9) | 157  (128−186) | − | 9.6  (6.1−13.1) | − | − | 279  (0−1485) | 5.3  (4.1−6.5) |  |
| Senegal,CLQ-R | 78 | 4.9  (3.8−6.0) | 245.1 (220.7−269.5) | 302  (131−473) | − | 14.3  (11.9−16.7) | − | − | 347  (0−1710) | 5.2  (3.6−6.8) |  |
| Gabon, CLQ-S | 8 | 1.5  (1.0−1.9) | 56  (36−75) | − | − | − | − | − | − | − |  |
| Gabon, CLQ-R | 51 | 3.3  (2.3−4.3) | 291  (243−339) | − | − | − | − | − | − | − |  |

PRN, pyronaridine; CLQ, chloroquine (S = susceptible, R = resistant); Q, quinine; MQ, mefloquine; AMQ, amodiaquine; APQ, amopyroquine; HF, halofantrine; PYR, pyrimethamine; ATM, artemether

^a^n = 29 for amodiaquine, n = 25 for pyrimethamine

^b^n = 6 for pyrimethamine

^c^Monodesethylamodiaquine
