# Supplementary material for: Review of pyronaridine anti-malarial properties and product characteristics
Source: Malar J. 2012 Aug 9;11:270. doi: 10.1186/1475-2875-11-270 (PMC3483207; doi:10.1186/1475-2875-11-270)
Supplement: Additional file 2 — Comparative IC50 for drug-resistant and -sensitive strains of P. falciparum. [file 1475-2875-11-270-S2.doc]

**Additional file 2.** Comparative IC_50_ for drug-resistant and -sensitive strains of *P. falciparum* .

| **Strain/clone[reference]** | | **IC_50_, nM** | | | | | | | | | | | | | | | | | | | | | |  |
| --- | --- | --- | --- | --- | --- | --- | --- | --- | --- | --- | --- | --- | --- | --- | --- | --- | --- | --- | --- | --- | --- | --- | --- | --- |
|  | **PRN** | | **CLQ** | **Q** | **MQ** | **AMQ** | **APQ** | **MEP** | | **HF** | | **PYR** | | **CYC** | **ART** | **DHA** | | **ATS** | **ATE** | | **ATM** | | **ATL** | |
| **Chloroquine-resistant** |  | |  |  |  |  |  |  |  | |  | |  | |  |  |  | |  |  | |  | |  |
| Tm90C2A (Thailand) | 2.51 | | 237.68 | − | − | − | − | − | − | | − | | − | | − | 2.81 | 9.97 | | − | − | | − | |  |
| T9.94 (Thailand) | 14.2 | | 31.5 | 310 | 17.5 | 18.3 | − | 17.8 | − | | − | | − | | − | − | − | | − | − | | − | |  |
| K1 (Thailand) | 3.94 | | 265.11 | − | − | − | − | − | − | | − | | − | | − | 2.35 | 4.14 | | − | − | | − | |  |
| K1 (Thailand) | 8.2 | | 214 | 461.8 | 22.8 | 11.2 | − | 39.9 | − | | − | | 7600 | | − | − | − | | − | − | | − | |  |
| K2 (Cambodia) | 7.19 | | 755 | 809 | 28.0 | − | − | − | 2.91 | | 26300 | | 22800 | | 14.1 | − | − | | 6.17 | 8.02 | | 13.8 | |  |
| K4 (Cambodia) | 7.04 | | 661 | 776 | 10.6 | − | − | − | 1.18 | | 23300 | | 25000 | | 11.4 | − | − | | 7.43 | 6.56 | | 9.82 | |  |
| K7 (Cambodia) | 5.35 | | 812 | 652 | 27.6 | − | − | − | 3.95 | | 6720 | | 13600 | | 19.0 | − | − | | 10.7 | 10.2 | | 19.0 | |  |
| K8 (Cambodia) | 8.18 | | 653 | 813 | 10.6 | − | − | − | 1.73 | | 17400 | | 5950 | | 6.12 | − | − | | 4.88 | 3.49 | | 7.06 | |  |
| K9 (Cambodia) | 7.07 | | 693 | 833 | 8.9 | − | − | − | 1.47 | | 26400 | | 1120 | | 8.10 | − | − | | 4.50 | 4.9 | | 8.78 | |  |
| K10 (Cambodia) | 6.33 | | 477 | 911 | 11.0 | − | − | − | 1.95 | | 7660 | | 14500 | | 10.8 | − | − | | 5.87 | 4.63 | | 6.99 | |  |
| K11 (Cambodia) | 7.16 | | 624 | 861 | 13.1 | − | − | − | 1.99 | | 11200 | | 4840 | | 9.48 | − | − | | 4.08 | 4.71 | | 5.39 | |  |
| K12 (Cambodia) | 7.28 | | 986 | 834 | 22.7 | − | − | − | 0.913 | | 11100 | | 10700 | | 13.3 | − | − | | 6.35 | 8.74 | | 10.8 | |  |
| K13 (Cambodia) | 11.0 | | 1160 | 811 | 14.0 | − | − | − | 1.77 | | 9740 | | 19500 | | 19.3 | − | − | | 7.32 | 7.75 | | 11.4 | |  |
| K14 (Cambodia) | 7.69 | | 417 | 369 | 4.6 | − | − | − | 1.02 | | 10700 | | 1120 | | 9.72 | − | − | | 5.58 | 5.99 | | 11.8 | |  |
| FCM6 (Cambodia) | 5.87 | | 1260 | 644 | 21.3 | − | − | − | 1.81 | | 16300 | | 16600 | | 15.2 | − | − | | 8.46 | 8.51 | | 7.97 | |  |
| VS1 (Vietnam) | 1.63 | | 419.98 | − | − | − | − | − | − | | − | | − | | − | 1.54 | 9.93 | | − | − | | − | |  |
| W2 (Indochina) | 15.4 | | 99.3 | 119 | 5.99 | 13.1 | − | − | − | | 51100 | | − | | − | − | − | | − | − | | − | |  |
| FCM29 (Africa) | 12.7 | | 1250 | − | − | 336^c^ | 34.5 | − | − | | − | | − | | − | − | − | | − | − | | − | |  |
| LS25 (Kenya) | 35.2 | | 368.0 | 673 | 60.9 | 31.2 | − | 67.2 | − | | − | | − | | − | − | − | | − | − | | − | |  |
| LS32 (Nigeria) | 21.6 | | 339.0 | 564 | 31.6 | 24.8 | − | 52.9 | − | | − | | − | | − | − | − | | − | − | | − | |  |
| FCR3 (The Gambia) | 2.21 | | 317.79 | − | − | − | − | − | − | | − | | − | | − | 1.64 | 5.82 | | − | − | | − | |  |
| LS33 (Ghana) | 5.4 | | 284.0 | 490 | 14.5 | 12.0 | − | 29.1 | − | | − | | − | | − | − | − | | − | − | | − | |  |
| D7 (Tanzania) | 18.0 | | − | − | − | − | − | − | − | | − | | − | | 76.0 | 23.0 | − | | − | 0.98 | | − | |  |
| LS20 (Zambia) | 11.0 | | 149.0 | 285 | 3.5 | 19.0 | − | 24.8 | − | | − | | − | | − | − | − | | − | − | | − | |  |
| LS21 (India) | 47.8 | | 286.5 | 520 | 15.2 | 35.8 | − | 66.4 | − | | − | | − | | − | − | − | | − | − | | − | |  |
| FCB (Colombia) | 1.53 | | 362.88 | − | − | − | − | − | − | | − | | − | | − | 2.63 | 7.40 | | − | − | | − | |  |
| **Chloroquine-susceptible** |  | |  |  |  |  |  |  |  | |  | |  | |  |  |  | |  |  | |  | |  |
| T9.96 (Thailand) | 3.7 | | 17.0 | 650 | 115.0 | 6.4 | − | 13.0 | − | | − | | − | | − | − | − | | − | − | | − | |  |
| L-3 (Africa) | 5.2 | | 28.0 | − | − | 13.1^c^ | 9.2 | − | − | | − | | − | | − | − | − | | − | − | | − | |  |
| D6^a^ (Africa) | 4.73 | | 6.60 | 45.5 | 43.9 | 9.74 | − | − | − | | 16.3 | | − | | − | − | − | | − | − | | − | |  |
| N (Nigeria) | 12.0 | | 20.1 | 159.5 | 28.1 | 16.3 | − | 17.9 | − | | − | | − | | − | − | − | | − | − | | − | |  |
| F32 (Tanzania) | 13.0 | | − | − | − | − | − | − | − | | − | | − | | 108 | 4.7 | − | | − | 3.2 | | − | |  |
| 7G8 (Brazil) | 13.0 | | − | − | − | − | − | − | − | | − | | − | | 45.0 | 7.5 | − | | − | 3.2 | | − | |  |
| Honduras^b^ (Honduras) | 4.4 | | − | − | − | − | − | − | − | | − | | − | | 3.2 | 7.0 | − | | − | 6.1 | | − | |  |
| H1 (Honduras) | 4.4 | | 14.4 | 122 | 27.2 | 8.5 | − | 9.1 | − | | − | | − | | − | − | − | | − | − | | − | |  |
| FC27(Papua New Guinea) | 14.5 | | 40.7 | 418 | 169.0 | 22.8 | − | 18.3 | − | | − | | − | | − | − | − | | − | − | | − | |  |
| NF54 (Amsterdam) | 1.9 | | 12.9 | 132 | 43.9 | 10.1 | − | 8.3 | − | | − | | − | | − | − | − | | − | − | | − | |  |
| 3D7 of N54 (unknown) | 2.31 | | 14.19 | − | − | − | − | − | − | | − | | − | | − | 2.22 | 9.39 | | − | − | | − | |  |

PRN, pyronaridine; CLQ, chloroquine; Q, quinine; MQ, mefloquine; AMQ, amodiaquine; APQ, amopyroquine; MEP, mepacrine; HF, halofantrine; PYR, pyrimethamine; CYC, cycloguanil; ART, artemisinin; DHA, dihydroartemisinin; ATS, artesunate; ATE, arteether; ATM, artemether; ATL, artelinate

^a^Mefloquine resistant

^b^Pyrimethamine resistant

^c^Monodesethylamodiaquine
