# Supplementary material for: Review of pyronaridine anti-malarial properties and product characteristics
Source: Malar J. 2012 Aug 9;11:270. doi: 10.1186/1475-2875-11-270 (PMC3483207; doi:10.1186/1475-2875-11-270)
Supplement: Additional file 3 — Acute toxicity studies with pyronaridine: summary of main findings. [file 1475-2875-11-270-S3.doc]

**Additional file 3.** Acute toxicity studies with pyronaridine: summary of main findings .

| **Model[reference]** | **Parameter** | **Effect (n/N animals)** | |
| --- | --- | --- | --- |
|  |  | **Pyronaridine** | **Chloroquine** |
| **Oral acute toxicity** | | | |
| Mouse | ED_50_, mg/kg ± SD | 6.8 ± 1.4 (n = 4) | 45.6 ± 6.3 |
|  | LD_50_, mg/kg ± SD | 1368 ± 238.9 | 663.4 ± 76.7 |
|  | Therapeutic index^a^ | 201.2 | 14.5 |
| Mouse | LD_50_, mg/kg ± SD | 1345 ± 175.5^b^ | 654.3 ± 88.3^b^ |
|  | 7-day mortality at 507 mg/kg dose (0.38 LD_50_) | 3.3% (1/30) | Not tested |
| Mouse | LD_50_, mg/kg [95% CI] | 1342 [1159−1553] (n = 10) | Not tested |
| Rat | LD_50_, mg/kg [95% CI] | 1281 [1163−1411] (n = 5) | Not tested |
| Rat | LD_50_, mg/kg | ≥2000 (n = 5 M and 5 F) | Not tested |
| Dog | 120 mg/kg (n = 2) or 240 mg/kg (n = 2) | Vomiting 4/4 dogs. Hyperemia of intestinal/gastric mucosa in 1/2 dogs autopsied | Not tested |
| Monkey | 240 mg/kg dose (60 mg/kg Day 1 then qd /2 days) | Elevated SGPT in 1/4 animals 20→107 IU/L, resolved ≤1 week | Not tested |
| **Intramuscular acute toxicity** | | | |
| Mouse | ED_50_, mg/kg ± SD | 4.97 ± 0.65 (n = 7) | 30.89 ± 5.8 |
|  | LD_50_, mg/kg ± SD | 250.6 ± 33.1 | 89.7 ± 34.0 |
|  | Therapeutic index^a^ | 50.1 | 2.9 |
| Rabbit | MLD^c^ | 80 mg/kg (1/5 deaths) | 20 mg/kg (1/4 deaths) |
|  | 80 mg/kg^c^ | 1/5 deaths, ECG changes | 3/3 deaths |
|  | 40 mg/kg | ECG changes, reversible bradycardia, 0/4 deaths | 1/5 deaths |
|  | 20 mg/kg | Prolonged QRS on ECG 0/5 deaths | 1/4 deaths, reversible bradycardia |
| Dog | MLD^d^ | 60 mg/kg (1/3 deaths) | 10 mg/kg (1/3 deaths) |
|  | 60 mg/kg^d^ | 1/3 deaths, 1 animal had clonic convulsion & vomiting & recovered next day, 1 had SGPT increased 22.5→85 IU/L, resolved ≤1 week. No further reports within 1-month follow up. | Not tested |
|  | 40 mg/kg | Well tolerated (n = 5) | Not tested |
|  | 20 mg/kg | Well tolerated (n = 5) | 2/2 deaths |
|  | 10 mg/kg | Not tested | 1/3 deaths, tremor and white foam spitting in survivors resolved by next day |
|  | 5 mg/kg | Not tested | Tremor & white foam spitting in 3/3 dogs resolved by next day |

^a^ED_50_/LD_50_

^b^Number of animals was not stated

^c^Data from the same animals

^d^Data from the same animals

ED_50_, dose at which agent displays 50% effectiveness; LD_50_, dose lethal to 50% of test population; MLD, minimal lethal dose; SGPT, serum glutamate pyruvate transaminase
