# Supplementary material for: Review of pyronaridine anti-malarial properties and product characteristics
Source: Malar J. 2012 Aug 9;11:270. doi: 10.1186/1475-2875-11-270 (PMC3483207; doi:10.1186/1475-2875-11-270)
Supplement: Additional file 4 — Sub-acute toxicity studies with pyronaridine: summary of main findings. [file 1475-2875-11-270-S4.doc]

**Additional file 4.** Sub-acute toxicity studies with pyronaridine: summary of main findings .

| **Model**  **[reference]** | **Parameter** | **Effect n/N animals** | |
| --- | --- | --- | --- |
|  |  | **Pyronaridine** | **Chloroquine** |
| **Oral sub-acute toxicity** | | | |
| Rat | 40 mg/kg/day for 14 days | 1/15 deaths, normal growth & development | Not tested |
|  | 100 mg/kg/day for 14 days | Not tested | 3/15 deaths, impaired growth and development |
|  | 200 mg/kg/day for 14 days | 3/15 deaths, impaired growth & development | Not tested |
|  | 23 mg/kg/day for 28 days | 0/10 deaths; yellow colouration of tissues; basophilic material in spleen | Not tested |
|  | 70 mg/kg/day for 28 days | 0/10 deaths; yellow skin colouration; increases in ALT and AST; mild decrease in AG ratio; yellow colouration of tissues; yellow/brown urine; spleen and lymph node enlargement; accumulation of basophilic material in many tissues usually accompanied by chronic inflammatory response although to a lesser degree than 210 mg/kg/day. | Not tested |
|  | 210 mg/kg/day for 28 days | 0/10 deaths; minor increases in peripheral neutrophil count and minor decreases in red blood cell parameters; increases in ALT and AST; small increases in BUN and total protein; mild decrease in AG ratio; yellow colouration of tissues; accumulation of basophilic material in many tissues (predominantly in spleen, bone marrow, liver and lungs) usually accompanied by chronic inflammatory response. | Not tested |
| Dog | 12 mg/kg/day for 30 days | 0/2 deaths. No abnormal laboratory or ECG changes | 2/2 deaths. Salivating, tremor, Vomiting and white foam spitting |
|  | 24 mg/kg/day for 30 days | 0/2 deaths. No abnormal laboratory or ECG changes | 2/2 deaths. Salivating, tremor, vomiting and white foam spitting |
|  | 5 mg/kg/day for 28 days | 0/6 deaths; loss of appetite, vomiting, soft stools and diarrhoea; yellow colouration of tissues; basophilic material but little or no inflammation | Not tested |
|  | 15 mg/kg/day for 28 days | 0/6 deaths; loss of appetite, vomiting, soft stools and diarrhoea; yellow colouration of tissues; decrease in platelets; basophilic material in many tissues, although to a lesser degree than 45 mg/kg/day; | Not tested |
|  | 45 mg/kg/day for 28 days | 0/6 deaths; loss of appetite, vomiting, soft stools and diarrhoea; weight loss and decrease in food consumption in females; yellow colouration of conjunctiva and tissues; moderate decreases in red blood cell parameters and mild decreases in platelets, increases in ALP, ALT and total protein; decreases in albumin and AG ratio; enlargement of mesenteric and submandibular lymph nodes; atrophy of testes, epididymides and prostate; accumulation of basophilic material associated with chronic inflammation in many tissues with liver and spleen most affected; hyperplasia of a number of tissues. | Not tested |
| **Intravenous sub-acute toxicity** | | | |
| Rabbit | 20 mg/kg/day Day 1, 10 mg/kg/day Days 2−7 & 30-day follow up | Well tolerated (n = 5) | 1/5 deaths |
