# Supplementary material for: Review of pyronaridine anti-malarial properties and product characteristics
Source: Malar J. 2012 Aug 9;11:270. doi: 10.1186/1475-2875-11-270 (PMC3483207; doi:10.1186/1475-2875-11-270)
Supplement: Additional file 5 — Efficacy of pyronaridine monotherapy in patients with P. falciparum malaria: studies conducted in China. [file 1475-2875-11-270-S5.doc]

**Additional file 5.** Efficacy of pyronaridine monotherapy in patients with *P. falciparum* malaria: studies conducted in China .

| **Reference** | **N** | **Treatment regimen, total dose /days^a^ (dose divided)** | **Time to fever clearance, h ± SD** | **Time to parasite clearance, h ± SD** | **Recrudescence^b^ at follow up, n/N (% Cure)** |
| --- | --- | --- | --- | --- | --- |
| **Oral** |  |  |  |  |  |
| Fu and Xiao, 1991; Shao, 1990 | 20 | 1200−1600 mg /3 | 39.5 | 64.8 | 4/12 (66.6) |
|  | 32 | 1200 mg /2 | 43.6 | 49.4 | 4/28 (85.7) |
|  | 20 | Control: CLR 1500 mg /3 | 53.4 | 45.6 | Not reported |
| Huang, et al. 1989 | 32 | 800 mg /2^c^ | 27.0 ± 14.1 | 57.2 ± 10.2 | 0/32 (100) |
|  | 32 | 1200 mg /3^d^ | 30.2 ± 13.8 | 57.9 ± 8.7 | 2/32 (93.8) |
| **Intramuscular** |  |  |  |  |  |
| Fu and Xiao, 1991; Shao, 1990 | 46 | 2 mg/kg /1 (bid) | 26.4 ±14.8 | 53.3± 15.6 | 4/30 (66.6) |
|  | 17 | 3 mg/kg /1 (bid) | 24.5 ± 13.5 | 51.3 ± 15.3 | Not reported |
|  | 135 | 4 mg/kg /1 (bid) | 24.8 ± 13.8 | 51.0 ± 11.9 | 2/31 (93.5) |
|  | 186 | 4 mg/kg /1 (bid) | range 19.0−35.0 | range 32.0−56.7 | Not reported |
| **Intravenous** |  |  |  |  |  |
| Fu and Xiao, 1991; Shao, 1990 | 22 | 2 mg/kg /1 (qd) | 27.2 ± 16.0 | 72.1 ± 25.5 | 4/6 (33.3) |
|  | 18 | 4 mg/kg /1 (bid or qd) | 19.4 | 53.6 | Not reported |
|  | 42 | 6 mg/kg /1 (bid or qd) | 27.8 ± 17.8 | 58.1 ± 14.7 | 0/4 (100) |

^a^Pyronaridine unless stated otherwise

^b^Cases of re-infection were not excluded

^c^Plain tablets: 500 mg Day 0, 300 mg Day 1

^d^Enteric-coated tablets

CLR, chloroquine
