# Supplementary material for: Review of pyronaridine anti-malarial properties and product characteristics
Source: Malar J. 2012 Aug 9;11:270. doi: 10.1186/1475-2875-11-270 (PMC3483207; doi:10.1186/1475-2875-11-270)
Supplement: Additional file 6 — Oral monotherapy with pyronaridine in the treatment of falciparum malaria: International studies. [file 1475-2875-11-270-S6.doc]

**Additional file 6.** Oral monotherapy with pyronaridine in the treatment of falciparum malaria: International studies .

| **Outcome** | **Adults** | | | | **Children** | |
| --- | --- | --- | --- | --- | --- | --- |
|  | **Ringwald *et al* (Cameroon)** | | **Looareesuwan *et al* (Thailand)** | | **Ringwald *et al* (Cameroon)** | |
|  | **Pyronaridine**  **32 mg/kg**  **/3 days^a^**  **(n = 40)** | **Chloroquine**  **25 mg/kg**  **/3 days^b^**  **(n = 41)** | **Pyronaridine**  **1200 mg**  **/3 days^c^**  **(n = 60)** | **Pyronaridine**  **1800 mg**  **/5 days^c^**  **(n = 26)** | **Pyronaridine**  **32 mg/kg**  **/3 days^a^**  **(n = 41)** | **Chloroquine**  **25 mg/kg**  **/3 days^b^**  **(n = 40)** |
| **Clinical response** |  |  |  |  |  |  |
| Treatment success, n (%)^d^ | 40 (100) | 24 (58.5) | 38 (63.3) | 23 (88.5) | 41 (100) | 24 (60.0) |
| Early treatment failure | 0 | 4 (9.8) | NA | NA | 0 | 11 (27.5) |
| Late treatment failure | 0 | 13 (31.7) | NA | NA | 0 | 5 (12.5) |
| **Parasitological response^e^** |  |  |  |  |  |  |
| A | 40 (100) | 18 (43.9) | NA | NA | 41 (100) | 21 (52.5) |
| B | 0 | 19 (46.3) | NA | NA | 0 | 14 (35.0) |
| C | 0 | 4 (9.8) | NA | NA | 0 | 5 (12.5) |
| **Fever clearance^f^** |  |  |  |  |  |  |
| Number with fever clearance, n (%) | 40 (100) | 24 (58.5) | NA | NA | NA | NA |
| Mean time to clearance, h ± SD  [range] | 33.5 ± 14.3  [24−72] | 40.0 ± 20.8  [24−96] | 84.0 ± 64.8  [8−316] | 81.4 ± 52.2  [12−200] | 35.7 ± 15.3  [NA] | 31.3 ± 15.2  [NA] |
| **Parasite clearance^f^** |  |  |  |  |  |  |
| Number with parasite clearance, n (%) | 40 (100) | 18 (43.9) | NA | NA | NA | NA |
| Mean time to clearance, h ± SD  [range] | 76.8 ± 14.6  [24−96] | 70.7 ± 14.9  [48−96] | 84.4 ± 22.1  [34−169] | 86.7 ± 24.4  [38−142] | 72.6 ± 15.6  [NA] | 70.9 ± 16.1  [NA] |

^a^16 mg/kg Day 0, 8 mg/kg Days 2 and 3

^b^10 mg/kg on Days 0 and 1, 5 mg/kg on Day 2

^c^Two doses of 300 mg on Day 0, one dose on each of the following treatment days

^d^Treatment success at Day 14 for Ringwald, *et al*. 1996 and 1988 and Day 28 for Looareesuwan *et al* 1996

^e^A = negative parasite count on or before Day 3 or positive on Day 3 but <25% of pre-treatment density plus negative thereafter until Day 14

B = positive on Day 3 under 25% of pretreatment density and either positive on Day 7 or alternative antimalarial was requested between Day 3 and 7

C = positive parasite count on Day 3 over 25% of pretreatment density or alternative anti-malarial required before Day 3.

^f^Fever and parasite clearance times are based on patients that had treatment success and parasitological response
