# Supplementary material for: Review of pyronaridine anti-malarial properties and product characteristics
Source: Malar J. 2012 Aug 9;11:270. doi: 10.1186/1475-2875-11-270 (PMC3483207; doi:10.1186/1475-2875-11-270)
Supplement: Additional file 7 — Efficacy of oral pyronaridine combination therapy in patients with P. falciparum malaria. [file 1475-2875-11-270-S7.doc]

**Additional file 7**

Efficacy of oral pyronaridine combination therapy in patients with *P. falciparum* malaria .

| **Reference** | **N** | **Treatment regimen, total dose, mgkg /days^a^** | **Mean time to fever clearance, h ± SD** | **Mean time to parasite clearance, h ± SD** | **Recrudescence^b^ at follow up, n/N (% Cure)** |
| --- | --- | --- | --- | --- | --- |
| **Pyronaridine plus sulphadoxine−pyrimethamine (Hainan Province)** | | | |  |  |
| Shao *et al* | 29 | 800+1000+50 /2 | 38.4 ± 20.1^c^ | 41.9 ± 9.7 | 0/20 (100) |
|  | 32 | 500+1500+75 /1 | 37.6 ± 19.6 | 46.6 ± 9.1 | 0/25 (100) |
|  | 40 | 500+1000+50 /1 | 30.1 ± 15.1 | 48.7 ± 9.9 | 0/32 (100) |
| Chen and Zheng | 44 | 1200+1000+25 /3 | 37.1 ± 17.8 | 40.9 ± 8.9 | 1/37 (97.3) |
|  | 33 | 900+1500+75 /3^d^ | 21.3 ± 12.7 | 71.1 ± 18.6 | 0/33 (100) |
|  | 40 | 800+100+50 /2 | 35 ± 12 | 49 ± 14 | 0/39 (100) |
|  | 39 | 800+1000+50 /2 | 30.3 ± 24.7 | 52.7 ± 15.8 | 0/37 (100) |
|  | 36 | 240^e^+1000+50 /2 | 38.8 ± 23.0 | 51.8 ± 14.4 | 0/35 (100) |
|  | 20 | 500+1500+75 /1 | 41.8 ± 20.9 | 46.5 ± 10.1 | 0/16 (100) |
|  | 41 | 500+1000+50 /1 | 37.0 ± 21.8^f^ | 47.3 ± 10.3^f^ | 0/16 (100) |
|  | 41 | 400+1000+50 /1 | 44.6 ± 22.8 | 51.6 ± 10.9 | 0/39 (100) |
|  | 30 | Control: S/P 1500+75 /1 | 58.6 ± 27.2^f^ | 53.3 ± 10.1^f^ | 6/25 (76.0) |
|  | 32 | Control: PRN 1200 /3 | 30.2 ± 13.8 | 57.9 ± 8.9 | 2/25 (92.0) |
| **Pyronaridine plus sulphadoxine−primaquine (Yunnan Province)** | | | | | |
| Chen and Zheng | 48 | 1200+1000+45 /2^d^ | 32.0 ± 16.3 | 58.7 ± 20.8 | 1/40 (97.5) |
|  | 34 | 1200+1500+67.5 /3 | 25.5 ± 19.1 | 58.0 ± 15.3 | 2/31 (93.5) |
|  | 32 | Control: PRN/S/P 800+1000+50 /2 | 37.3 ± 17.6 | 59.8 ± 18.9 | 4/30 (86.7) |
| **Pyronaridine plus nitroquine (Hainan Province)** | | | | | |
| Chen *et al* | 11 | 600+25 | 33.0 ± 30.3 | 53.1 ± 18.5 | 2/11 (81.8) |
|  | 43 | 800+40 | 31.1 ± 15.0 | 45.8 ± 5.3 | 7/43 (83.7) |
|  | 31 | 800+80 | 35.0 ± 17.3 | 48.7 ± 12.1 | 4/31 (87.1) |
|  | 33 | Control: PQ/NQ 750+50 | 38.7 ± 18.3 | 49.5 ± 12.8 | 9/33 (72.7) |
|  | 7 | Control: PQ/NQ 975+32.5 | 47.7 ± 23.5 | 56.7 ± 20.5 | 3/7 (57.1) |
|  | 42 | Control: PRN 1200 | 32.9 ± 12.4 | 48.3 ± 23.3 | 5/42 (88.1) |
|  | 5 | Control: PQ 750 | 46.3 ± 53.0 | >122.0 ± 47.9 | 4/5 (20) |
| **Pyronaridine plus dihydroartemisinin** | | | | | |
| Liu *et al* | 32 | 800+300 /3^g^ | 35.7 ± 24.7 | 23.8 ± 10.1 | 0 (100) |
|  | 25 | Control: PRN 1600 /3^d^ | 35.8 ± 16.5 | 49.4 ± 20.3 | 0 (100) |
|  | 24 | Control: DHA 640 /7^d^ | 52.6 ± 38.9 | 22.9 ± 6.5 | 1/24 (95.8) |
| **Pyronaridine plus artesunate (co-formulation)** | | | | | |
| Ramharter *et al* | 14 | Mean dose 6.3+2.1 (ratio 6:2)/3 tablet | median 16 h | – | 0/11 (100)^h^ |
|  | 15 | Mean dose 9.9+3.3 (ratio 9:3)/3 tablet | median 16 h | – | 0/13 (100)^h^ |
|  | 15 | Mean dose 13.2/4.8 (ratio 12:4)/3 tablet | median 8 h | – | 0/15 (100)^h^ |
|  | 15 | Mean dose 11.4+3.8 (ratio 9:3)/3 granules | median 8 h | – | 0/14 (100)^h^ |

| Tshefu *et al* [121] |  | Pyramax (pyronaridine plus  artesunate 180:60‑mg) tablets  Range:7.2:2.4 mg/kg to 13.8:4.6 mg/kg  N= 849 Pyramax | 7.9 h | 23.9 h | 4/784 (99.5)^i^ |
| --- | --- | --- | --- | --- | --- |
|  |  |  |  |  |  |
| Rueangweerayut *et al* |  | Pyramax (pyronaridine plus  artesunate 180:60‑mg) tablets  Range:7.1:2.4 mg/kg to 14.0:4.7 mg/kg  N= 848 Pyramax | 15.7 h^j^ | 31.7 h^j^ | 6/749 (99.2)^i^ |

^a^Pyronaridine+sulphadoxine+pyrimethamine, pyronaridine+sulphadoxine+primaquine, pyronaridine+nitroquine or pyronaridine+dihydroartemisinin dose in mg as sub-titled unless stated otherwise.

^b^At Day 28, cases of re-infection were not excluded

^c^N = 27

^d^Two doses given on Day 0

^e^Pyronaridine given IM

^f^*P* < 0.001 for fever clearance and *P* < 0.05 for time to aparasitaemia

^g^400/100 mg pyronaridine+dihydroartemisinin on Day 0, 400+100 mg on Days 1 and 2.

CLR, chloroquine; DHA, dihydroartemisinin; NQ, nitroquine; P, pyrimethamine; PQ, piperaquine; PRN, pyronaridine; PRQ, primaquine; S sulphadoxine

^h^Cure at Day 28, PCR-corrected (i.e. cases of reinfection were excluded)

^i^PCR corrected ACPR (efficacy evaluation population)

^j^ Includes data from Cambodia

−, not reported in paper
