# Supplementary material for: Review of pyronaridine anti-malarial properties and product characteristics
Source: Malar J. 2012 Aug 9;11:270. doi: 10.1186/1475-2875-11-270 (PMC3483207; doi:10.1186/1475-2875-11-270)
Supplement: Additional file 8 — Clinical studies conducted in China of pyronaridine alone and in combination with primaquine in patients with P. vivax malaria. [file 1475-2875-11-270-S8.doc]

**Additional file 8.** Clinical studies conducted in China of pyronaridine alone and in combination with primaquine in patients with *P. vivax* malaria .

| **Reference** | **N** | | **Treatment regimen, total dose /days^a^** | | **Time to fever clearance, h ± SD** | **Time to parasite clearance, h ± SD** | **Recrudescence^b^ at follow up, n/N (%)** |
| --- | --- | --- | --- | --- | --- | --- | --- |
| **Oral** | | | | | | | |
| Shao, Fu and Xiao | 99 | | 1200 mg /1 (tid) | | 22.7 ± 48.0  (range 22−48) | 41.7 ± 72.0  (range 41−72) | NA |
|  | 5 | | 1200 mg /2 | | 36.2 | 41.5 | 0/5 (0) |
|  | 19 | | 1200 mg /3 | | 18.0 | 58.9 | 0/19 (0) |
|  | 37 | | 1200 mg /3 | | 25.5 ± 4.2 | 59.3 ± 17.5 | 2/37 (5.4) |
|  | 20 | | Control: CHL 1200 mg /3 | | 28.1 ± 8.1 | 60.0 ± 17.2 | 1/20 (5.0) |
| Shao | 50 | | 1600 mg /3 | | NA | 13−18 | NA |
| **Intramuscular** |  | |  | |  |  |  |
| Shao, Fu and Xiao | 25 | | 2 mg/kg /1 (bid) | | 19.6 ± 4.2 | 47.5 ± 17.2 | 3/16 (18.8) |
|  | 53 | | 4 mg/kg /1 (bid) | | 22.6 ± 13.9 | 43.3 ± 10.8 | 4/29 (13.8) |
|  | 207 | | 4 mg/kg /1 (bid) | | range 17.0−26.3 | range 28.7−43.6 | NA |
| Fu and Xiao | 71 | | 4 mg/kg /1 (bid) | | 23.7 | 40.4 | 4/19 (21.1) |
| **Intravenous** |  | |  | |  |  |  |
| Shao, Fu and Xiao | 14 | | 2 mg/kg /1 (bid) | | 20.3 ± 7.8 | 60.1 ± 29.3 | 2/10 (20.0) |
|  | 13 | | 6 mg/kg /1 qd or bid | | 21.3 ± 9.8 | 50.7 ± 21.2 | 0/7 (0) |
| **Pyronaridine plus primaquine (oral)** | | | | |  |  |  |
| Huei *et al* | | 86 | | PRN/PRQ 1200+90 mg /3 | NA | NA | 3/86 (3.5) |
|  | | 444 | | PRN/PRQ 1600+90 mg /3 | NA | NA | 9/444 (2.0) |
|  | | 126 | | Control: CHL/PRQ 1200 mg /3 + 180 mg /8 | NA | NA | 1/126 (0.8) |
| Liu *et al* | | 236 | | PRN/PRQ 1600+120 mg /4 | NA | NA | 20/236 (8.5) |
|  | | 241 | | Control: CHL/PRQ 1500+120 mg /4 | NA | NA | 30/241 (12.4) |
|  | | 228 | | Control: CHL/PRQ 1500 mg /4+ 180 mg /8 | NA | NA | 8/228 (3.5) |

^a^Pyronaridine unless stated otherwise

^b^Cases of re-infection were not excluded

CLR, chloroquine; NA, not available; PRN, pyronaridine; PRQ, primaquine
