# Supplementary material for: Review of pyronaridine anti-malarial properties and product characteristics
Source: Malar J. 2012 Aug 9;11:270. doi: 10.1186/1475-2875-11-270 (PMC3483207; doi:10.1186/1475-2875-11-270)
Supplement: Additional file 9 — Treatment-emergent adverse events with pyronaridine oral monotherapy in the treatment of falciparum malaria. [file 1475-2875-11-270-S9.doc]

**Additional file 9.** Treatment-emergent adverse events with pyronaridine oral monotherapy in the treatment of falciparum malaria .

| **Adverse events, n (%)** | **Adults** | | | | **Children** | |
| --- | --- | --- | --- | --- | --- | --- |
|  | **Ringwald *et al* (Cameroon)** | | **Looareesuwan *et al*  (Thailand)** | | **Ringwald *et al* (Cameroon)** | |
|  | **Pyronaridine**  **32 mg/kg**  **/3 days^a^**  **(n = 40)** | **Chloroquine**  **25 mg/kg**  **/3 days^b^**  **(n = 41)** | **Pyronaridine**  **1200 mg**  **/3 days^c^**  **(n = 69)** | **Pyronaridine**  **1800 mg**  **/5 days^c^**  **(n = 32)** | **Pyronaridine**  **32 mg/kg**  **/3 days^a^**  **(n = 41)** | **Chloroquine**  **25 mg/kg**  **/3 days^b^**  **(n = 40)** |
| Headache | 2 (5.0) | 0 | (38) | (36) | 4 (9.8) | 1 (2.5) |
| Dizziness | 1 (2.5) | 1 (2.4) | (28) | (33) | 2 (4.9) | 1 (2.5) |
| Nausea | 4 (10.0) | 5 (12.2) | (18) | (12) | 2 (4.9) | 4 (10.0) |
| Vomiting | 2 (5.0) | 2 (4.9) | (11) | (13) | 3 (7.3) | 3 (7.5) |
| Abdominal pain | 13 (32.5) | 2 (4.9) | (8) | (5) | 9 (22.0) | 5 (12.5) |
| Diarrhoea | 10 (25.0) | 1 (2.4) | (11) | (7) | 5 (12.2) | 1 (2.5) |
| Palpitation | 2 (5.0) | 2 (4.9) | − | − | 3 (7.3) | 1 (2.5) |
| Pruritus | 7 (17.5) | 18 (43.9) | − | − | 2 (4.9) | 19 (47.5) |
| Cutaneous eruption | 1 (2.5) | 0 | − | − | − | − |
| Hypoacusia | 1 (2.5) | 0 | − | − | − | − |

^a^16 mg/kg Day 0, 8 mg/kg Days 2 and 3

^b^10 mg/kg on Days 0 and 1, 5 mg/kg on Day 2

^c^Two doses of 300 mg on Day 0, one dose on each of the following treatment days

−, not reported in paper
