# Supplementary material for: Review of pyronaridine anti-malarial properties and product characteristics
Source: Malar J. 2012 Aug 9;11:270. doi: 10.1186/1475-2875-11-270 (PMC3483207; doi:10.1186/1475-2875-11-270)
Supplement: Additional file 10 — Treatment-emergent adverse events with fixed-dose pyronaridine-artesunate in the treatment of falciparum malaria in children. [file 1475-2875-11-270-S10.doc]

**Additional file 10.** Treatment-emergent adverse events with fixed-dose pyronaridine-artesunate in the treatment of falciparum malaria in children

| **Pyronaridine-artesunate dose/ formualtion:** | **6:2 mg/kg tablets**  **(N = 14)** | **9:3 mg/kg tablets**  **(N = 15)** | **12:4 mg/kg tablets**  **(N = 15)** | **9:3 mg/kg granules**  **(N = 15)** |
| --- | --- | --- | --- | --- |
| Serious adverse event | 2 (14) | 0 | 0 | 0 |
| Any adverse event | 13 (93) | 11 (73) | 13 (97) | 12 (90) |
| Infection | 9 (64) | 8 (53) | 10 (67) | 11 (73) |
| Gastrointestinal disorder | 7 (50) | 3 (20) | 3 (20) | 1 (7) |
| Headache | 5 (36) | 1 (7) | 2 (13) | 1 (7) |
| Fatigue or pyrexia | 2 (14) | 2 (13) | 1 (7) | 2 (13) |
| Cough | 2 (14) | 1 (7) | 2 (13) | 1 (7) |
| Splenomegaly of hepatomegaly | 1 (7) | 2 (13) | 3 (20) | 1 (7) |
| Anorexia | 2 (14) | 0 | 1 (7) | 1 (7) |
| Drug-related adverse event | 5 (36) | 4 (27) | 5 (33) | 3 (20) |
| Gastrointestinal disporders | 3 (21) | 2 (13) | 2 (13) | 1 (7) |
| Fatigue or pyrexia | 1 (7) | 1 (7) | 0 | 1 (7) |
| Splenomegaly or hepatomegaly | 0 | 1 (7) | 2 (13) | 0 |
| Anaemia | 1 (7) | 0 | 0 | 0 |
| Anorexia | 1 (7) | 0 | 0 | 1 (7) |
| Headache | 1 (7) | 0 | 0 | 1 (7) |
| Hyperhidrosis | 0 | 0 | 1 (7) | 0 |

All values are shown as n (%)
